# The First Noncovalent-Bonded Supramolecular Frameworks of (Benzylthio)Acetic Acid with Proline Compounds, Isonicotinamide and Tryptamine

**DOI:** 10.3390/molecules27238203

**Published:** 2022-11-24

**Authors:** Justyna Sienkiewicz-Gromiuk, Aleksandra Drzewiecka-Antonik

**Affiliations:** 1 Department of General and Coordination Chemistry and Crystallography, Institute of Chemical Sciences, Faculty of Chemistry, Maria Curie-Sklodowska University in Lublin, M. Curie-Sklodowska Sq. 2, 20-031 Lublin, Poland; 2 Institute of Physics, Polish Academy of Sciences, Aleja Lotnikow 32/46, 02-668 Warsaw, Poland

**Keywords:** (benzylthio)acetic acid, proline, isonicotinamide, tryptamine, supramolecular assemblies, crystal structure, noncovalent interactions, infrared spectra, thermal behaviour

## Abstract

The co-crystallization of (benzylthio)acetic acid (HBTA) with L-proline (L-PRO), D-proline (D-PRO), DL-proline (DL-PRO), isonicotinamide (INA) and tryptamine (TPA) led to the formation of five novel crystalline compounds: L-PRO^±^·HBTA (**1**), D-PRO^±^·HBTA (**2**), DL-PRO^±^·HBTA (**3**), INA·HBTA (**4**) and TPA^+^·BTA^−^ (**5**). The prepared supramolecular assemblies were characterized by single crystal X-ray diffraction, an elemental analysis, FT-IR spectroscopy and a thermal analysis based on thermogravimetry (TG) combined with differential scanning calorimetry (DSC). Additionally, their melting points through TG/DSC measurements were established. All fabricated adducts demonstrated the same stoichiometry, displayed as 1:1. The integration of HBTA with selected N-containing co-formers yielded different forms of multi-component crystalline phases: zwitterionic co-crystals (**1**–**3**), true co-crystal (**4**) or true salt (**5**). In the asymmetric units of **1**–**4**, the acidic ingredient is protonated, whereas the corresponding N-containing entities take either the zwitterionic form (**1**–**3**) or remain in the original neutral figure (**4**). The molecular structure of complex **5** is occupied by the real ionic forms of both components, namely the (benzylthio)acetate anion (BTA^−^) and the tryptaminium cation (TPA^+^). In crystals **1**–**5**, the respective molecular residues are permanently bound to each other via strong H-bonds provided by the following pairs of donor···acceptor: O_carboxylic_···O_carboxylate_ and N_pyrrolidinium_···O_carboxylate_ in **1**–**3**, O_carboxylic_···N_pyridine_ and N_amine_···O_carboxylic_ in **4** as well as N_indole_···O_carboxylate_ and N_aminium_···O_carboxylate_ in **5**. The crystal structures of conglomerates **1**–**5** are also stabilized by numerous weaker intermolecular contacts, including C–H···O (**1**–**3**, **5**), C–H···S (**1**, **2**, **5**), C–H···N (**5**), C–H···C (**5**), C–H···π (**1**–**5**) as well as π···π (**4**) interactions. The different courses of registered FT-IR spectral traces and thermal profiles for materials **1**–**5** in relation to their counterparts, gained for the pure molecular ingredients, also clearly confirm the formation of new crystalline phases.

## 1. Introduction

The rational design and synthesis of new supramolecular crystalline systems, with specific crystal networks and physiochemical properties based on the skilful use of noncovalent interactions present between molecules, is broadly understood as the paramount objective of crystal engineering [1,2]. The creation of stable, supramolecular frameworks is accomplished with the aid of complementary noncovalent intermolecular interactions, encompassing a wide range of contacts, both attractive and repulsive in nature, mainly including hydrogen bonds, aromatic π···π stacking interactions, and electrostatic and charge-transfer attractions [3,4,5]. Hydrogen bonding, being a directional and the strongest force, occupies a unique position among all available intermolecular non-bonded contacts occurring between the constituent species in supramolecular architectures [6]. The targeted patterns of binding between the functional components in multi-component crystals must contain at least one set of noncovalent interactions with a noticeably higher strength than that of the others [7,8]. The presence of hydrogen bonds is therefore necessary in the process of the controlled aggregation of molecular components into solid supramolecular assemblies [6]. The geometrical and spatial interlinking of intermolecular interactions within the structure of supramolecules generates special structural units named supramolecular synthons [9], described by Etters’s graph-set notation [10]. The supramolecular synthons act indeed as the robust and transferable connectors utilized in the durable and predictable linking of molecular tectons within supramolecules [8,9]. The crystals of supramolecular complexes consist of at least two distinct molecular ingredients present in the crystal lattice. The different molecular components included in one crystalline material may exist as an ion (charged residue), a solvent (the neutral liquid residue) or a co-former (the neutral solid residue). Depending on the residue types present in the crystal lattice, the multi-component crystalline solids have been classified into three main categories: molecular salts, co-crystals and solvates [11]. Moreover, seven subclasses arise from the main categories which represent the possible compositions of the crystalline forms of supramolecular systems: three of them, designated as true salts, true co-crystals and true solvates, are strictly derived from the main classes, whereas the remaining four, defined as salt solvates, co-crystal solvates, co-crystal salts and co-crystal salt solvates, are hybrids of the main categories [12]. In the true co-crystal subclass, composed of neutral molecules of at least two different molecular ingredients, only a particular form defined as a zwitterionic co-crystal is clearly distinguishable. Unlike true co-crystals with a classical composition, zwitterionic co-crystals contain at least one co-former in the form of a dipolar ion presented by a molecule having both positively and negatively charged functional sites. The global net charge of zwitterionic co-crystals is ultimately zero as is the case of true co-crystals composed of neutral residues only [13].

**Figure 1:**
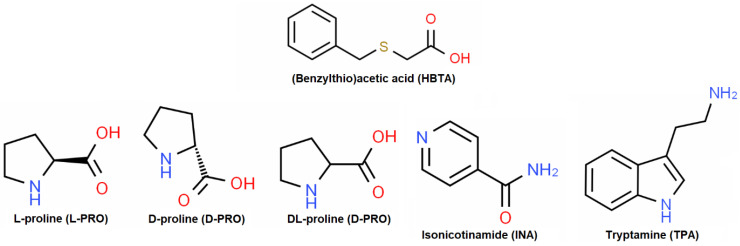
The chemical diagrams of the (benzylthio)acetic acid (HBTA) and selected N-containing co-formers.

The carboxylic derivatives containing additionally aromatic/cyclic rings composed of mono- or heteroatoms and other potential binding sites, such as NH_2_, OH, SO_2_, NO_2_, halogens, N, O, S, and CH/CH_2_/CH_3_, act as excellent and versatile functional components in the creation of supramolecular architectures, as they provide various kinds of hydrogen bond acceptors and/or donors and the π-conjugated system for extending their networks [14]. According to the principles of crystal engineering, (benzylthio)acetic acid (HBTA) fits perfectly into the process of supramolecular complexation due to its chemical structure (Figure 1), which is composed of the following moieties: a carboxylic group, two CH_2_ entities, an organic sulphide –S– species and an aryl unit.

As arises from the crystalline structure of single-component HBTA crystals [15], all types of functional groups are involved in the formation of intermolecular interactions, including the strong O–H···O and weak C–H···O, C–H···S and C–H···π contacts that stabilize the three-dimensional supramolecular framework. The ability to form intermolecular interactions by individual functional groups of HBTA was also verified on the basis of molecular electrostatic potential (MEP) as well as electrostatic potential (ESP) surfaces [16], as MEP and ESP descriptors are beneficial tools for defining the regions where the compound can have noncovalent interactions [17]. The MEP and ESP results revealed that the carboxylic group is the most privileged fragment of the HBTA molecule. The most negative region reflected on the MEP surface associated with the oxygen atoms within the carboxylic species confirmed that the hydrogen bonding, involving the hydroxyl oxygen as the strongest donor and the carbonyl oxygen as the strongest acceptor, gives the energetically preferred O–H···O and C–H···O hydrogen bonding present in the crystal structure of HBTA. Moreover, the most positive potential located on the acidic proton makes the O–H···O hydrogen bond the easiest to form. At the same time, O–H···O hydrogen bonding is much more stable compared to other types of noncovalent interactions generated by the HBTA molecule, which was reflected in the much shorter donor–acceptor distance. The less negative MEP and ESP region accumulated on the sulphur atom indicates its role as an acceptor in weak C–H···S noncovalent interactions. For the MEP surface in dimer structure of HBTA, the most positive charge located on one proton of each of the methylene groups indicates, in turn, that methylene carbons operate as weak polar donors in C–H···O and C–H···π noncovalent contacts [15,16]. The only known crystal structures obtained with the participation of the HBTA agent are four metal–organic coordination polymers with selected transition metal cations, namely Co (II) [18], Cu (II) [19], Cd (II) and Zn (II) [20]. The HBTA reagent occurs in these crystals as fully deprotonated BTA^−^ anions forming primarily coordination bonds with the metal centres only through the carboxylate oxygens (Cu (II) and Zn (II) complexes) or with the simultaneous partnership of sulphur (Co (III) and Cd (II) compounds).

**Figure 2:**
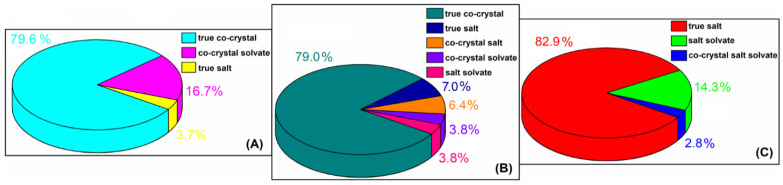
The percentage distribution of the reported forms of carboxylic acid:PRO; (**A**) carboxylic acid: INA; (**B**) and carboxylic acid: TPA (**C**) supramolecular associations [21,22].

The eponymous N-containing species (Figure 1), such as L-proline (L-PRO), D-proline (D-PRO), DL-proline (DL-PRO), isonicotinamide (INA) and tryptamine (TPA), are also useful in making organic crystalline solids by various noncovalent interactions due to the possession of diverse functional units. The chemical structures of the ingredients in question influences their intensive exploitation as molecular building blocks in the construction of a broad family of hydrogen-bonded supramolecular aggregates, especially with organic acids, as confirmed by numerous CSD [21] results obtained by using the *ConQuest2022.1.0* program [22]. There is only one monoclinic *P2_1_/c* [23,24] variant of the DL-PRO racemate as well as the orthorhombic *P2_1_2_1_2_1_* [25,26,27] and triclinic *P-1* [26] polymorphic forms of the L-PRO enantiomer. To date, the racemates and enantiomers of proline as co-crystallization agents form **54** different carboxylic:PRO crystal structures [21,22]. The vast majority of them belong to the true co-crystals group but co-crystal solvates and true salts are also observed (Figure 2A). INA, which is well-known and has been intensively investigated due to its rich hydrogen bonding behaviour, possesses as many as seven polimorphic forms. Four of the five monoclinic variants show the same *P2_1_/c* [28,29,30] symmetry, while only one crystallizes as the *Pc* [29] polymorph. Apart from the monoclinic varieties, the single-component crystals of INA also create two different orthorhombic polymorphs defined as *Pbca* [31] and *Pca2_1_* [32]. Due to its considerable synthon flexibility as well as conformational flexibility [28] in an active manner that causes supramolecular reactions with a wide variety of organic acids, the INA component yielded **186** crystal structures [21,22] of carboxylic acid:INA supramolecular complexes. The carboxylic acid:INA aggregates exhibit the greatest diversity of solid crystalline phases. Their disclosed molecular structures indicate they belong to the true co-crystal, true salt, co-crystal salt, co-crystal solvate and salt solvate groups (Figure 2B). Pure TPA exists only in one orthorhombic *P2_1_2_1_2_1_* phase [33]. Among **35** reported organic crystal structures [21,22] synthesized by combining the tryptamine with carboxylic acids, over 97% are salts which also include the incorporated solvent’s molecules (Figure 2C).

This work presents an exploration of the ability of the HBTA component to supramolecular complexation via the participation of co-formers in the form of enantiomeric and racemic proline compounds, isonicotinamide and tryptamine (Figure 1). The discussed topic is novel in terms of the application of an HBTA agent as the effective structural unit in the construction of organic supramolecules, since reports in the literature have not revealed any data related to supramolecular complexes based on this acidic component up till now. This publication focuses primarily on presenting the crystal structures of the first crystalline multi-component phases obtained from the HBTA molecular brick along with the identification of the noncovalent forces’ networks generated via the coupling of classical hydrogen bonds and other weak noncovalent interactions. In addition to the structural and supramolecular context, the TG/DSC thermal and infrared spectral characterization of produced crystalline solid forms is also considered.

## 2. Results and Discussion

**Figure 3:**
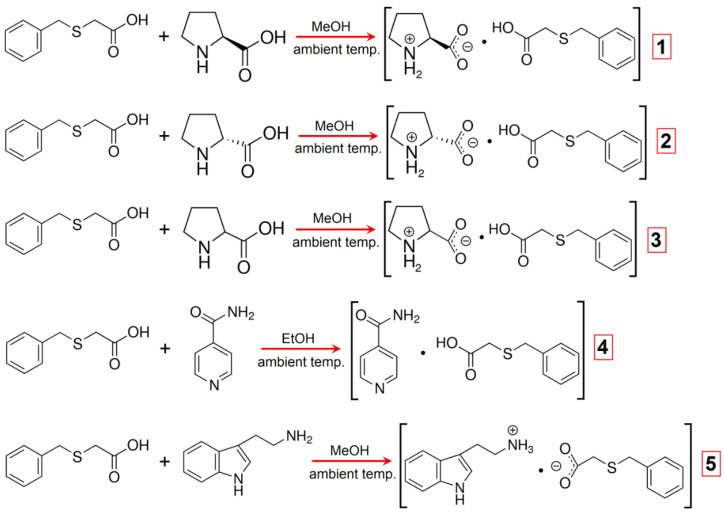
Synthetic route for the preparation of supramolecular complexes **1**–**5**.

All of the noncovalent-bonded supramolecular architectures, **1**–**5**, were prepared by using the co-crystallization process that entailed mixing the corresponding N-containing ingredients with (benzylthio)acetic acid (HBTA) at a 1:1 ratio. Well-shaped crystals were then isolated under ambient conditions utilizing the natural solvent-evaporating technique with the addition of short-chained alcohols, due to the similar and high levels of solubility of the title building units in given alcohols. The synthetic pathway for the production of the adducts **1**–**5** is depicted in Figure 3, while the procedure of their synthesis is described in detail in the Materials and Methods section. The grown crystals of aggregates **1**–**5** were subjected to the SC X-ray diffraction measurements. All complexes crystallized without solvent molecules. The revealed structures showed that the co-crystallization products are identified as zwitterionic co-crystals (**1**–**3**), true co-crystals (**4**) and true salts (**5**). The obtained multicomponent crystalline phases were also analysed with FT-IR spectroscopy as well as the thermal analysis.

### 2.1. Crystal Structures Descriptions

The X-ray crystallographic study of the five new structures of S-containing carboxylic acid, namely (benzylthio)acetic acid (HBTA) with L-proline (**1**), D-proline (**2**), DL-proline (**3**), isonicotinamide (**4**) and tryptamine (**5**), is described below. The analysed structures are designated as follows: L-PRO^±^·HBTA (**1**), D-PRO^±^·HBTA (**2**), DL-PRO^±^·HBTA (**3**), INA·HBTA (**4**) and TPA^+^·BTA^−^ (**5**). The summary of crystal data, experimental details and refinement results of structures in question as well as the geometry of intermolecular interactions in the crystals **1**–**5** are shown in Table 1 and Table 2, respectively.

#### 2.1.1. Zwitterionic Co-Crystals from (Benzylthio)Acetic Acid and Proline Compounds (**1**–**3**)

**Figure 4:**
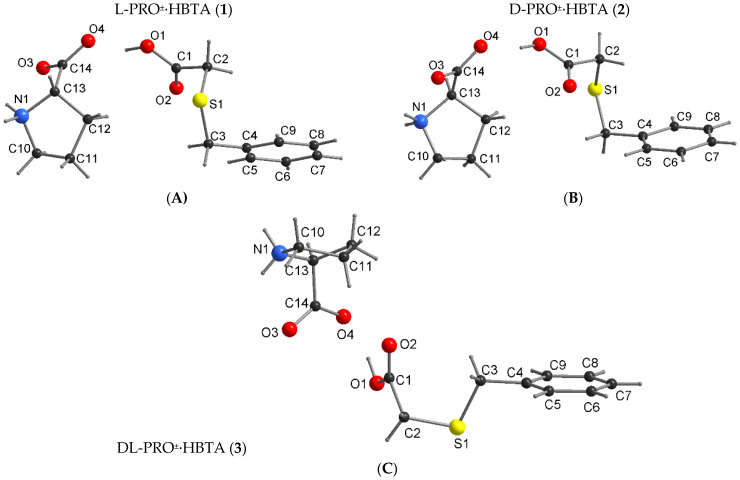
The molecular structure of zwitterionic co-crystals **1** (**A**), **2** (**B**) and **3** (**C**) with atom-numbering scheme.

The enantiomeric adducts **1** and **2** crystallize in a monoclinic crystal system with the same space group *P2_1_* and a similar unit cell dimension (Table 1). The atom numbering and the conformation of **1** and **2** adopted in the crystal are shown in Figure 4. Their asymmetric unit consists of an independent protonated HBTA component and an L-PRO^±^ or D-PRO^±^ prolinium zwitter ion with a 1:1 stoichiometry (Figure 4). The designated C‒O bond lengths (Table S1) support the occurrence of the neutral HBTA molecules (C1‒O1: 1.329 (6) Å (in **1**) and 1.333 (4) Å (in **2**); C1‒O2:1.205 (5) Å (in **1**) and 1.207 (4) Å (in **2**)) as well as carboxylate groups within both L-PRO^±^ (C14‒O3: 1.249 (4) Å; C14‒O4: 1.252 (3) Å) and D-PRO^±^ (C14‒O3: 1.251 (5) Å; C14‒O4: 1.260 (3) Å) zwitterion ions indicating the formation of L-PRO^±^·HBTA (**1**) and D-PRO^±^·HBTA (**2**) zwitterionic co-crystals, not salts.

The bond lengths and angles of the prolinium ions and acid molecules of **1** and **2** (Table S1) are within the expected ranges and are equal within the experimental error. The value of dihedral angle between the plane of the phenyl ring of HBTA and the pyrrolidine moiety is 69.4 (3)° for **1** and **2**, respectively. Apart from many similarities between the presented co-crystals, there is a difference in the conformation of the acid component (see Figure 4). The torsion angles C1‒C2‒S1‒C3/C2‒S1‒C3‒C4, describing the stereochemistry of the alkyl sulfanyl chain of acid molecule, are 71.1 (7)°/59.1 (8)° for L-PRO^±^·HBTA and −70.9 (1)°/−58.8 (1)° for D-PRO^±^·HBTA. The absolute values of the torsion angles are the same, within the experimental error, and the difference in the sign does not affect the crystal structure of either of the compounds, which are analogous.

**Figure 5:**
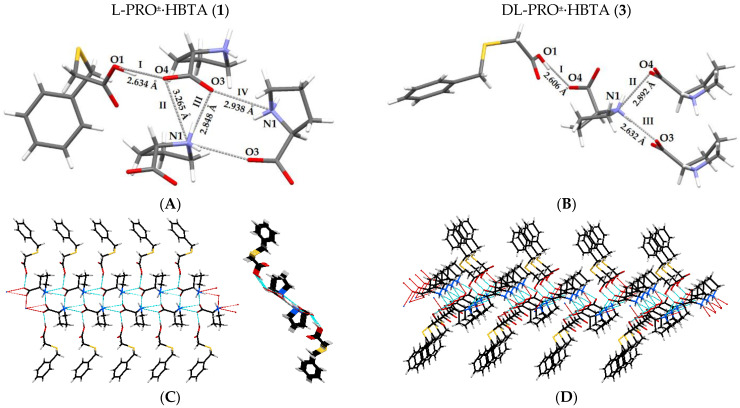
H-bonded synthons in the crystal lattice of **1** (**A**) and **3** (**B**). H-bonded ribbon along the *a* axis and its cross section observed in **1** (**C**) and 2D network in crystal **3** (**D**).

**Figure 6:**
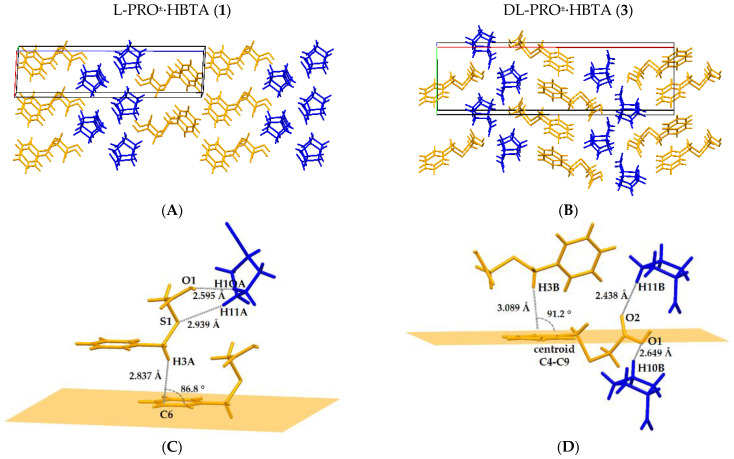
Comparison of crystal packing of **1** (**A**) and **3** (**B**) (orange = HBTA, and blue = PRO^±^) with geometric presentation of weak contacts in crystal **1** (**C**) and **3** (**D**).

The pattern of hydrogen bond interactions is the same for both of the discussed co-crystals (Table 2) and is presented in Figure 5A. The molecule of (benzylthio)acetic acid is bonded to the prolinium zwitterion ion via an O1‒H1O1···O4 strong hydrogen bond (Synthon I, Figure 5A). The PRO^±^ itself is further associated with two other prolinium zwitterion ions by three intermolecular N‒H···O bonds (Synthons II-IV, Figure 5A). These hydrogen bonding interactions link the molecules into a one-dimensional infinite ribbon, with the prolinium ions inside and the acid molecules outside, as presented in Figure 5C. The weak C‒H···O and C‒H···S contacts (Table 2), in which the molecules of PRO^±^ act as donors and the O/S atoms of the HBTA molecules serve as hydrogen-bond acceptors, as well as the head-to-tail C3–H···π interactions between the acid molecules, stabilized the three-dimensional structure of the analysed zwitterionic co-crystals (Figure 6A,C).

DL-proline and (benzylthio)acetic acid also form a 1:1 co-crystal **3** (Figure 4). It crystallizes in the orthorhombic space group *Pna2_1_*. The C‒O bond distances (Table S1) of the HBTA molecule (C1‒O1: 1.334 (3) Å; C1‒O2:1.203 (3) Å) and the carboxylate groups within the second component (C14‒O3: 1.250 (3) Å; C14‒O4: 1.265 (3) Å) indicate the formation of DL-PRO^±^·HBTA zwitterionic co-crystals. The values of all bond lengths and angles (Table S1) are similar to those observed for co-crystals **1** and **2**. The differences are observed in the orientation of the pyrrolidine ring in respect to the phenyl ring. These are quite close to being parallel (as opposed to the more perpendicular orientations for **1** and **2**) and the dihedral angle is equal to 15.7 (5)°. The use of a racemic proline for the synthesis of co-crystals resulted in significant changes in the conformation of the acid molecule (Figure 4, C1‒C2‒S1‒C3/C2‒S1‒C3‒C4 torsion angles are 58.7 (4)°/172.4 (3)°) in relation to that previously observed in co-crystals with L- and D-proline enantiomers. These changes generated a different pattern of intermolecular interactions in co-crystal **3**. In the prolinium ions, the NH_2_^+^ moiety, being a hydrogen bond donor, and a carboxylate group, serving as an acceptor, form the intramolecular N1‒H···O3 hydrogen bond, closing the five-membered ring. The interaction of the DL-prolinium zwitterion ion with (benzylthio)acetic acid takes place through O1‒H1O1···O4 contact (Synthon I, Figure 5B), with similar bond lengths and angles as those determined for co-crystals **1** and **2** (Table 2). Furthermore, the NH_2_^+^ moiety of DL-PRO^±^ acts as a hydrogen donor to two other DL-prolinium zwitterion ions, forming synthon II (N1‒H···O4) and synthon III (N1‒H···O3), as presented in Figure 5B.

The crystal packing pattern of **3** differs from that observed for the co-crystal of (benzylthio)acetic acid with L-proline and D-proline. The described supramolecular synthons in DL-PRO^±^·HBTA form a 2D network, as shown in Figure 5D, made of parallel ribbons similar to those observed for co-crystal **1**. Moreover, the weak C–H···π interaction between acid molecules and the C‒H···O contact between DL-PRO^±^ and HBTA were determined (Table 2) and they stabilize the 3D supramolecular architecture of co-crystal **3** (Figure 6B,D). The crystallographic unique molecules form an ABAB motif, whereas for co-crystal **1**, an AABB arrangement of molecules was observed (Figure 6).

#### 2.1.2. True Co-Crystal of (Benzylthio)Acetic Acid and Isonicotinamide (**4**)

**Figure 7:**
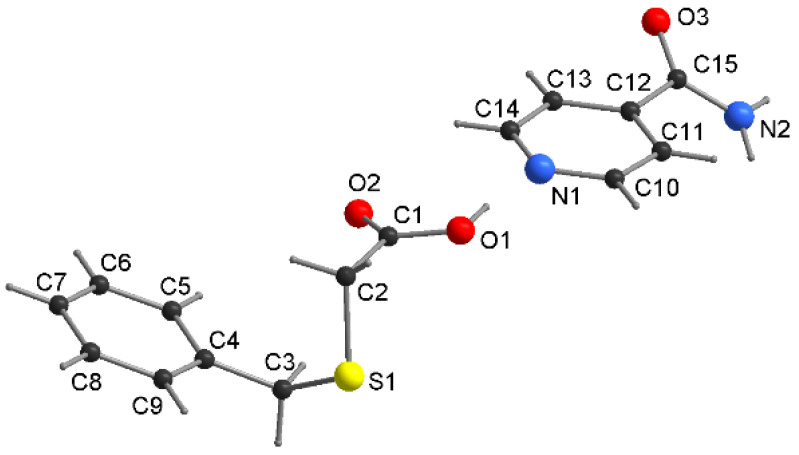
The molecular structure of **4** with atom-numbering scheme.

The crystal structure analysis of **4** reveals a 1:1 co-crystal (INA·HBTA; Figure 7) that crystallizes in a triclinic space group *P-1*. The length of the C1–O1, C1–O2 bonds in the acid molecule are 1.297 (2) Å and 1.208 (2) Å (Table S1), indicating that the hydrogen atom is located on the O1 atom. The conformation of the acid molecule is similar to that observed for co-crystal **2** (the torsion angles: C1–C2–S1–C3/C2–S1–C3–C4 are −75.5 (1)°/−56.5 (1)°), and the dihedral angle between the plane of the phenyl ring and the pyridine moiety is 15.3 (2)°.

**Figure 8:**
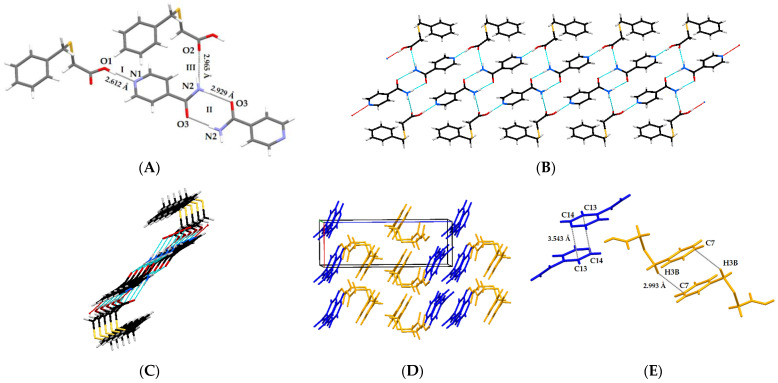
Three different heterosynthons in co-crystal **4** (**A**); H-bonded infinite ribbon of **4** along the *a* axis (**B**) and its cross section (**C**); packing diagram along *b* axis in crystal **4** in the AABB manner (orange = HBTA and blue = INA) (**D**); π···π stacking between INA molecules and C–H···π interactions between HBTA molecules (**E**).

The interaction of the HBTA acid with INA takes place through an O1–H1···N1 hydrogen bond (Synthon I, Figure 8A). Two more hydrogen-bonded synthons are observed in the isonicotinamide co-crystal (Figure 8A). The donation of hydrogen atoms from the NH_2_ part of the carboxamide group of INA to the carbonyl moiety (*a*) of other INA molecules (N2–H1···O3) and of (*b*) carboxylic acid forms (*a*) a homomeric R^2^_2_ (8) dimer (Synthon II) and (*b*) a linear N2–H2···O2 interaction (synthon III), respectively. In crystals, these supramolecular hydrogen bonds generate an infinite ribbon (Figure 8B,C) that is further stabilized by the head-to-head π···π stacking between the INA molecules and the head-to-tail C–H···π interactions between the HBTA molecules forming a 3D, hydrogen-bonded network (Figure 8D,E).

#### 2.1.3. True Salt Formed from (Benzylthio)Acetic Acid and Tryptamine (**5**)

**Figure 9:**
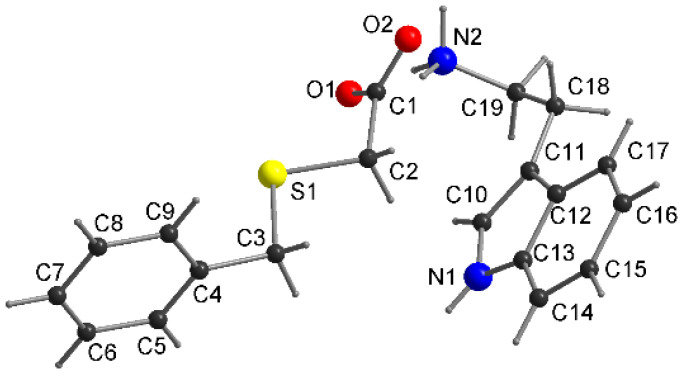
The molecular structure of **5** with atom-numbering scheme.

Compound **5** crystallizes in the monoclinic *P2_1_* space group with one molecule of each component in the asymmetric unit (Figure 9). The Fourier analysis showed that there is a proton transfer from the carboxyl group of the acid molecule to the NH_2_ moiety of tryptamine, indicating the formation of a salt: TPA^+^·BTA^−^. The C1–O1/O2 bond distances of 1.260 (3) and 1.250 (3) Å in the BTA^−^ molecule (Table S1) also confirmed the presence of an ionized carboxylic group. The alkyl sulfanyl chain of the HBTA molecule is planar (C1–C2–S1–C3/C2–S1–C3–C4 torsion angles are equal −179.7 (4)°/173.3 (4)°) and the phenyl ring C4–C9 is twisted 73.4 (3)° to the indole ring.

**Figure 10:**
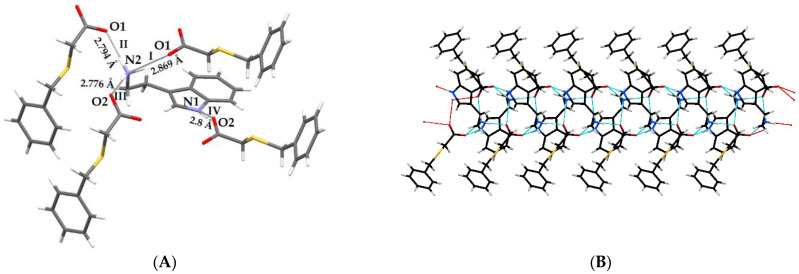

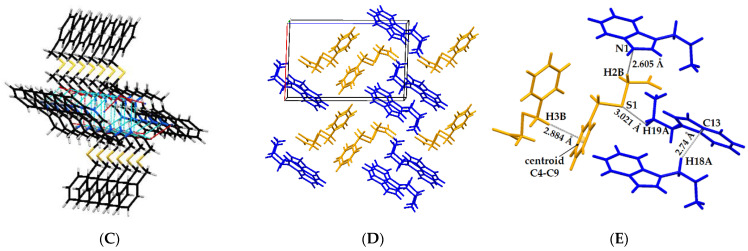
Strong H-bond interactions between TPA^+^ and BTA^−^ (**A**); H-bonded infinite ribbon along the *a* axis (**B**) and its cross section (**C**); packing diagram along *b* axis (orange = BTA^−^ and blue = TPA^+^) (**D**); weak C–H···N/S and C–H···π interaction (**E**) in the crystal lattice of **5**.

In crystals, the aminium NH_3_^+^ moiety of one molecule of TPA^+^ acts as a donor, whereas the O atoms of the carboxylate groups of three BTA^−^ molecules become hydrogen-bond acceptors (Figure 10A, synthons I-III). The synthon IV, observed in salt **5**, formed on the basis of the interaction of the indole nitrogen N1 of the tryptaminium cation and the carboxylate acceptor O2 of the (benzylthio)acetate anion (Figure 10A). Those well-directed hydrogen bonds led to the formation of a supramolecular ribbon, as shown in Figure 10B,C, which is additionally enhanced by the C–H···O, C–H···N and C–H···S contacts (Table 2). Moreover, the weak head-to-tail C–H···π interaction between the TPA^+^ cations as well as the head-to-tail C–H···π contact between the BTA^−^ anions become important in stabilization of the 3D supramolecular architecture of salt **5** (Figure 10D,E).

### 2.2. FT-IR Spectral Analysis

**Figure 11:**
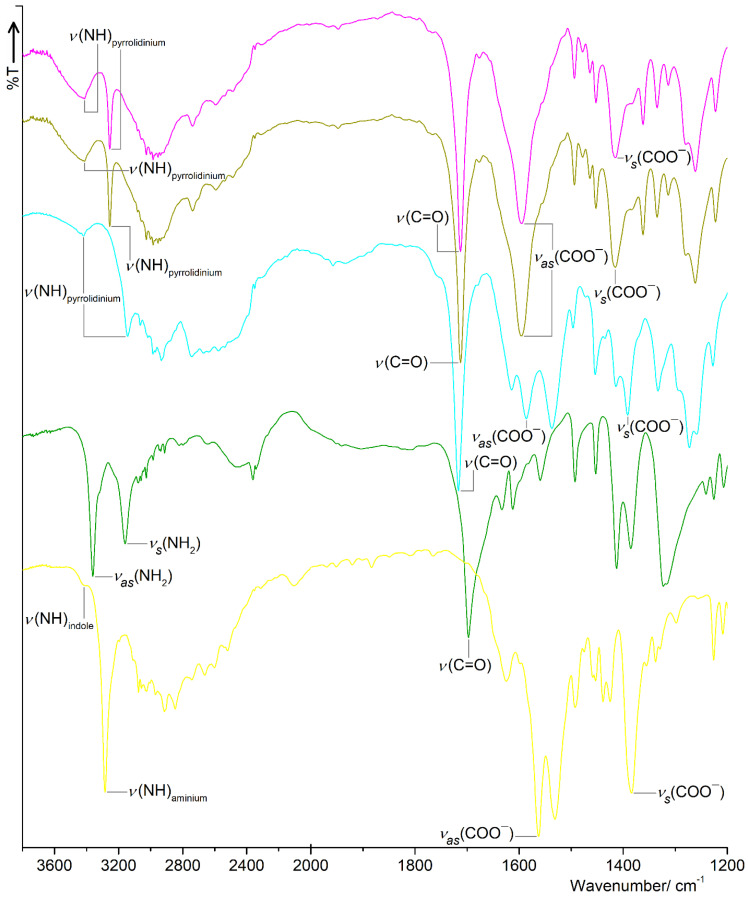
FT-IR spectra for associations **1**–**5** in the range of 3800–1200 cm^−1^.

Fourier transformed infrared (FT-IR) spectroscopy is a valuable technique for analysing the structure of solid materials based on the identification of the various functional groups present in the tested samples; therefore, this analytical method is also an excellent approach to the characterization and study of the solid crystalline phases of co-crystallization products. The FT-IR spectral signatures of the starting components and the supramolecular aggregates in question were performed in order to ascertain the crystalline form of received solid phases **1**–**5** in the context of co-crystal/salt formation. The solid-state FT-IR spectra of complexes **1**–**5** covering the most diagnostic spectral range between 3800 cm^−1^ and 1200 cm^−1^ are presented in Figure 11, whereas the FT-IR profiles showing the comparison of recorded spectral traces for the complexes and the corresponding molecular components in the entire spectral range are depicted in Figure S1. 

The formation of new products **1**–**5** is reflected by the presence of vibrational bands corresponding to the functional groups derived from both co-formers. Additionally, the generation of new crystalline phases **1**–**5** is also demonstrated by the occurrence of the appropriate shifts of characteristic stretching vibrations in the FT-IR spectra of adducts **1**–**5** in comparison to their positions shown in the spectra of the starting species. The crystal structures of adducts **1**–**5** are established via various energetic H-bonds, among which the privileged ones are those formed by the strong functional groups, such as COO^−^/COOH, NH_3_^+^/amide as well as the pyrrolidinium/pyridine/indole nitrogen atom, respectively; hence, the strongest shifts of stretching vibrations are related to these beneficial functional groups.

The key H-bonding functional group in the HBTA agent is the acidic moiety. The most specific vibrational mode of the organic acid function is attributed to the carbonyl stretching *ν* (C=O) of the COOH group. The absorption band associated with this vibrational mode for free HBTA agents is located at 1697 cm^−1^ (KBr pellet) [16] or 1694 cm^−1^ (ATR mode) [15], and is characterized by the highest activity in the HBTA spectral profile. In case of adducts **1**–**4**, the most intense peaks, situated at 1712 cm^−1^ (**1** and **2**), 1716 cm^−1^ (**3**) and 1699 cm^−1^ (**4**), respectively, are connected with the carbonyl stretches originating from the acidic unit. These results point to the fact that the HBTA molecular ingredient after co-crystallization remains in its neutral and protonated form. The observed shifts in the free acid absorption band towards higher wave numbers (higher energies) confirm that the complexes **1**–**4** crystallize in the form of co-crystals, which is consistent with X-ray crystal structures. In the spectra of complexes **1**–**3**, the two absorption bands, corresponding to the stretching *ν_as_* (COO^−^) and *ν_s_* (COO^−^) vibrations derived from the negatively charged carboxylate group of the zwitterionic form of proline compounds, are also observed. The peaks connected with the stretching *ν_as_* (COO^−^) and *ν_s_* (COO^−^) vibrations of the deprotonated carboxylate group of L-PRO^±^, D-PRO^±^ and DL-PRO^±^ zwitter ions are located at 1596 cm^−1^ and 1415 cm^−1^ for complexes **1** and **2**, as well as 1586 cm^−1^ and 1391 cm^−1^ for aggregate **3**, respectively, which is in line with the spectral data obtained for zwitterionic co-crystals with phenylboronic and 4-iodophenylboronic acids [34]. The zwitterionic form of proline compounds also contains the positively charged NH_2_^+^ moiety resulting from the proton transfer from the acidic part to the pyrrolidine nitrogen atom within the molecule, so the two high-energy stretching *ν* (NH) vibrations of the protonated NH_2_^+^ group appeared as a relatively broad and uniquely strong band [35]. In the spectral plots of materials **1**–**3**, the absorption bands related to the stretching *ν* (NH) vibrations came from the NH_2_^+^ group and are situated at 3414 cm^−1^ and 3255 cm^−1^ for enantiomers **1** and **2** as well as 3418 cm^−1^ and 3143 cm^−1^ for racemate **3**.

The free INA ingredient shows the carbonyl stretching *ν* (C=O) vibrational mode at 1681 cm^−1^ (*ν* (C=O): 1667 cm^−1^ [36]). As results from the spectral trace of aggregate **4**, only one distinct band is observed near 1700 cm^−1^. This most intensive absorption peak located at 1699 cm^−1^ is probably attributed to both the stretching *ν* (C=O) vibrational mode of the protonated HBTA unit as well as the *ν* (C=O) vibrational fundamental of the amide part of INA, due to their participation in the formation of the hydrogen bonds. The absorption bands of stretching *ν_as_* (NH_2_) and *ν_s_* (NH_2_) vibrations of the primary amine from INA are found in the FT-IR profile of complex **4** at 3361 cm^−1^ and 3160 cm^−1^, respectively, which is in agreement with the spectral data obtained for free INA (*ν_as_* (NH_2_): 3370 cm^−1^ and *ν_s_* (NH_2_): 3186 cm^−1^ [36]) and for the supramolecular framework of INA with L-malic acid (*ν_as_* (NH_2_): 3383 cm^−1^ and *ν_s_* (NH_2_): 3159 cm^−1^ [37]).

In the infrared spectrum of adduct **5**, the carbonyl stretching *ν* (C=O) absorption band of the acid disappeared completely and was replaced by new characteristic absorption bands of stretching *ν_as_* (COO^−^) and *ν_s_* (COO^−^) vibrations found at 1562 cm^−1^ and 1384 cm^−1^, respectively, which confirms the occurrence of the carboxylate group belonging to the deprotonated anionic form of the HBTA component. The neutral molecule of the tryptamine agent consists of the aliphatic ethylamino chain attached to the indole core. From the viewpoint of supramolecular complexation, the most important absorption bands in the infrared trace of TPA are connected with the stretching vibrations *ν_as_* (NH_2_) and *ν_s_* (NH_2_) of the amine moiety as well as the stretching vibrational mode *ν* (NH) of the indole unit. The *ν_as_* (NH_2_) and *ν_s_* (NH_2_) fundamental modes are active in the TPA spectral profile at 3344 cm^−1^ and 3283 cm^−1^, respectively, which are in accordance with the vibrational data obtained for 5-hydroxytryptamine (*ν_as_* (NH_2_): 3318 cm^−1^ and *ν_s_* (NH_2_): 3272 cm^−1^) [38]. The TPA component also shows the indole *ν*(NH) stretching mode at 3418 cm^−1^, which is in line with the wavenumber (*ν* (NH): 3419 cm^−1^) found for the basic indole system [39]. The absence of the absorption bands corresponding to the *ν_as_* (NH_2_) and *ν_s_* (NH_2_) in the spectral signature of complex **5** with the simultaneous presence of an absorption peak attributed to the stretching fundamental *ν* (NH) of the indole at the same position indicates that, during the co-crystallization process, the acidic proton was directed into the amine, not the indole, nitrogen atom of tryptamine, resulting in the formation of the aminium NH_3_^+^ group. The intense absorption band seen at 3285 cm^−1^ corresponds to the stretching vibrations of the H-bonded NH_3_^+^ moiety. What is more, the extended absorption, involving numerous combination bands and overtones, to about 2000 cm^−1^ with a clearly noticeable peak located at 2106 cm^−1^ as well as the strong band of the symmetric deformation vibrations observed at 1531 cm^−1^, also confirm the existence of NH_3_^+^ species in the structure of adduct **5** [40]. The fact of formation of the tryptaminium cation (TPA^+^) and (benzylthio)acetate anion (BTA^−^) is corroborated with the crystal structure of complex **5**, defining this solid phase as a true salt.

### 2.3. Thermal Analysis in Air

A thermal analysis as an important tool for determining the thermal stability of various classes of materials because it provides information about the possible presence of solvent molecules in their structures as well as the mass loss and peak values related to the exothermic and/or endothermic effects occurring at different temperature ranges [41].

**Figure 12:**
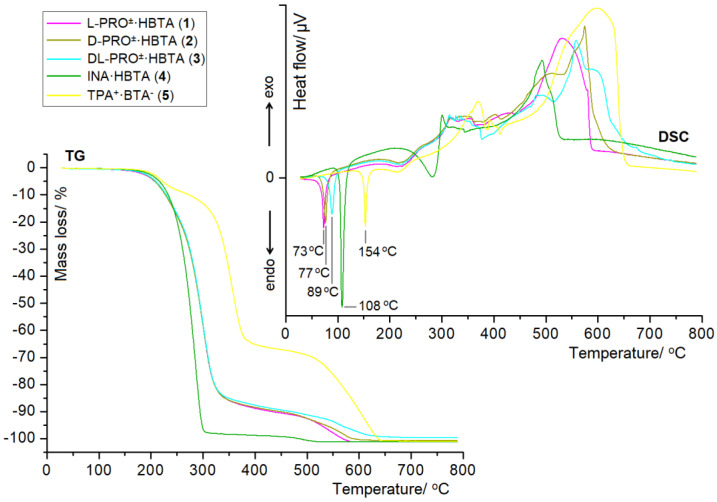
TG and DSC thermograms for complexes **1**–**5**.

The thermal behaviour of prepared solid supramolecular associations was investigated by simultaneously employing thermogravimetry (TG) and differential scanning calorimetry (DSC). The TG and DSC traces registered for all adducts are illustrated in Figure 12, while the comparison of the TG and DSC thermal curves between the complexes and the free building bricks is shown in Figure S2. The plateaus visible on the TG profiles up to about 100 °C indicate the unsolvated nature of all the supramolecular frameworks. The sharp DSC endothermic dips situated at 73 °C (**1**), 77 °C (**2**), 89 °C (**3**), 108 °C (**4**) and 154 °C (**5**) correspond to the melting points of the obtained aggregates. The DSC results revealed that the melting points of all the adducts are distinct from HBTA (64 °C) [15] and the corresponding N-containing co-formers (L-PRO: 234 °C; D-PRO: 227 °C; DL-PRO: 213 °C; INA: 159 °C; TPA: 117 °C), which actually indicates the formation of new crystalline phases. The zwitterionic co-crystals of L-PRO^±^·HBTA (**1**), D-PRO^±^·HBTA (**2**) and DL-PRO^±^·HBTA (**3**) exhibit melting points in between the two components (Figure S2A–C), thus the materials **1**–**3** have a much lower thermal stability compared to that of the starting proline agents and a slightly higher thermal stability than that of HBTA. The melting point of the true co-crystal INA·HBTA (**4**), also found in between the two ingredients (Figure S2D), implies that the INA·HBTA (**4**) displays a decrease in the thermal stability of isonicotinamide and an improvement in the thermal stability of HBTA. The true salt TPA^+^·BTA^−^ (**5**) shows a noticeably higher thermal stability than that of the tryptamine and HBTA (Figure S2E), which is due to its higher melting point than that of the initial components. The TG and DSC curves show that all supramolecular adducts decompose only after melting. Among the first exothermic effects reflected on the DSC traces for complexes **1**–**5**, the one located at a much higher temperature than the others is the exothermic peak with a maximum at 370 °C, which was observed for salt **5**. The most intensive exothermic peaks visible on the DSC profiles of compounds **1**–**5** are situated around 531 °C, 574 °C, 558 °C, 483 °C and 598 °C, respectively, indicating the dominant stage of the decomposition process of these substances. It is evident from the TG curves that all of the complexes **1**–**5** decompose completely without any solid residues.

## 3. Materials and Methods

### 3.1. Materials

All co-formers and solvents were sourced from commercial products with analytical grades and used without further purification. (Benzylthio)acetic acid (HBTA) and L-proline were provided by Sigma-Aldrich. D-proline was obtained from Reanal Laboratory Chemicals Ltd. whereas the DL-proline was sourced from Tokio Chemical Industry Co., Ltd. (TCI). Isonicotinamide was received from Alfa Aesar-Thermo Fisher Scientific while tryptamine was purchased from Acros Organics B.V.B.A. Solvents in the form of methanol and ethanol were received from Avantor Performance Materials Poland S.A. (formerly POCH S.A.).

### 3.2. Preparation of the Solid Forms ***1**–**5***

Co-crystallization process was applied in order to produce good crystals quality of synthesized supramolecular associations. Well-shaped single crystals of adducts **1**–**5** were harvested as a result of engagement of slow solvent evaporation technique from methanolic or ethanolic solution, containing equimolar amounts of HBTA and respective N-containing entities at room temperature.

The details of synthesis, the colours and shapes of collected crystals, yield of co-crystallization experiments, and the results of elemental analysis performed for complexes **1**–**5** are presented below:

**L-PRO^±^·HBTA** (**1**): HBTA (0.364 g, 2 mmol) and L-PRO (0.230 g, 2 mmol) were dissolved separately in 5 mL of MeOH each. Both solutions were then combined and stirred for 5 min. The resulting solution was allowed to evaporate naturally at ambient temperature and after a few days, light-brown, plate-like crystals were obtained.

Yield for **1**: 0.545 g (91.75%); Elemental analysis results for **1** (C_5_H_9_NO_2_^±^·C_9_H_10_O_2_S) Calcd (%): C, 56.55; H, 6.44; N, 4.71; S, 10.78. Found (%): C, 56.28; H, 6.31; N, 4.85; S, 10.93.

**D-PRO^±^·HBTA** (**2**): HBTA (0.364 g, 2 mmol) was dissolved in 5 mL MeOH. D-PRO (0.230 g, 2 mmol), also dissolved in 5 mL of MeOH, was added to this solution. After several days, by slow evaporation of the solvent at room temperature, light-brown, cube-shaped crystals were obtained.

Yield for **2**: 0.567 g (95.45%); Elemental analysis results for **2** (C_5_H_9_NO_2_^±^·C_9_H_10_O_2_S) Calcd (%): C, 56.55; H, 6.44; N, 4.71; S, 10.78. Found (%): C, 56.37; H, 6.29; N, 4.89; S, 10.97.

**DL-PRO^±^·HBTA** (**3**): HBTA (0.182 g, 1 mmol) and DL-PRO (0.115 g, 1 mmol) was dissolved separately in 5 mL of MeOH each. Next, the solutions were mixed together and stirred for a few minutes. Light-brown, block-shaped crystals were harvested after allowing the final mixture to evaporate at ambient temperature for a few days.

Yield for **3**: 0.269 g (90.57%); Elemental analysis results for **3** (C_5_H_9_NO_2_^±^·C_9_H_10_O_2_S) Calcd (%): C, 56.55; H, 6.44; N, 4.71; S, 10.78. Found (%): C, 56.71; H, 6.37; N, 4.63; S, 10.69.

**INA·HBTA** (**4**): An ethanolic solution (5 mL) of INA (0.122 g, 1 mmol) was added to an ethanolic solution (5 mL) of HBTA (0.182 g, 1 mmol). Next, the combined solution was stirred for a while and left to evaporate at room temperature, producing light-brown, plate-like crystals after several days.

Yield for **4**: 0.285 g (93.75%); Elemental analysis results for **4** (C_6_H_6_N_2_O·C_9_H_10_O_2_S) Calcd (%): C, 59.19; H, 5.30; N, 9.20; S, 10.53. Found (%): C, 58.84; H, 5.18; N, 9.03; S, 10.79.

**TPA^+^·BTA^−^** (**5**): The methanolic solution (5 mL) of HBTA (0.182 g, 1 mmol) was mixed with the methanolic solution (5 mL) of TPA (0.160 g, 1 mmol). The obtained solution was stirred for 5 min and left to evaporate for a few days at ambient temperature, resulting in a growth of needle-like crystals of brown colour.

Yield for **5**: 0.301 g (88.01%); Elemental analysis results for **5** (C_10_H_13_N_2_^+^·C_9_H_9_O_2_S^−^) Calcd (%): C, 66.64; H, 6.48; N, 8.18; S, 9.36. Found (%): C, 66.80; H, 6.65; N, 8.07; S, 9.54.

### 3.3. Physical Measurements

The determination of the weight percentages of carbon, hydrogen, nitrogen and sulphur content in the supramolecular assemblies **1**–**5** was performed by means of a Perkin Elmer 2400 Series II CHNS/O elemental analyser (PerkinElmer Inc., Waltham, MA, USA), utilizing the CHNS mode of operation.

The infrared spectra of supramolecular complexes **1**–**5** and free components were recorded on FT/IR-4600 (JASCO Corporation, Tokyo, Japan) FT-IR spectrometer utilizing the KBr pellet technique in the spectral range between 4000 and 400 cm^−1^.

The thermal behaviour of multicomponent crystalline products and free co–formers were investigated on the basis of the thermogravimetry (TG) coupled with differential scanning calorimetry (DSC). The TG/DSC measurements were conducted on a SETSYS 16/18 (Setaram, Caluire, France) thermal apparatus with the dynamic purge of air 0.75 dm^3^ h^−1^. Weights of 8.149 to 8.770 mg of tested samples placed in the alumina crucibles were heated over the temperature range of 30–800 °C at a constant heating rate of 10 °C min^−1^.

### 3.4. Single-Crystal X-ray Diffraction Study

The crystallographic measurements of multi-component crystals **1**–**5** were performed on an Oxford Diffraction Xcalibur CCD diffractometer (Oxford Diffraction Ltd., Abington, UK) with graphite-monochromated MoK*_α_* radiation (λ = 0.71073 Å). The diffraction data for adducts **1**, **2** and **4** were collected at 293 (2) K while the data sets for complexes **3** and **5** were gathered at 100 (2) K and 120 (2) K, respectively, using the *ω* scan technique with an angular scan width of 1.0°. The *CrysAlis PRO* v. 1.171.37.35g (Agilent Technologies Ltd., Oxford, UK) and *CrysAlis PRO* v. 1.171.39.46 (Rigaku Oxford Diffraction, Oxford, UK) software packages [42,43] were applied for data collection, refinement of cell dimensions, data reduction and multi-scan absorption correction. The structures’ solution and refinement were carried out using the direct methods in *SHELXS*-86 [44] and by full-matrix least-squares on *F*^2^ using *SHELXL*-2018/3 [45] within the *WinGX 2021.1* software [46]. The non-hydrogen atoms were refined anisotropically. The H-atoms attached to the carbon atoms were positioned geometrically, whereas the H-atoms attached to the oxygen and nitrogen atoms were located from the different Fourier maps and refined isotropically. The *Mercury 2022.1.0* program [47] was exploited for the molecular and crystal structures visualizations along with the exploration of crystal packing as well as for analysis of intermolecular interactions.

## 4. Conclusions

(Benzylthio)acetic acid (HBTA) was successfully applied as a co-former in the preparation of the first five noncovalent-bonded supramolecular associations with proline (PRO) compounds, isonicotinamide (INA) as well as tryptamine (TPA). The co-crystallization experiments, depending on the type of incorporated N-containing species, contributed to the generation of diverse crystalline phases of the supramolecular materials in question as zwitterionic co-crystals (L-PRO^±^·HBTA, D-PRO^±^·HBTA, DL-PRO^±^·HBTA; **1**–**3**), true co-crystals (INA·HBTA, **4**) and true salts (TPA^+^·BTA^−^, **5**). The individual building units present in crystal structures **1**–**5** are dominantly bound with each other with the COOH···O (**1**–**3**), COOH···N and NH_2_···COOH (**4**) or NH_3_^+^···COO^−^ and N_indole_H···COO^−^ (**5**) synthons, which show the importance of these interactions in the formation of the co-crystals or salts under investigation, respectively. The analysed structures show some similarities. The ribbon patterns, with the N-containing component inside and the acid molecules outside, were observed. The distinct geometries of the N-containing species as well as the different sets of weaker intermolecular contacts, such as C–H···O, C–H···S, C–H···N, C–H···C, C–H···π, and π···π, have resulted in the construction of varied 3D supramolecular architectures, in which the crystallographically unique molecules form AABB or ABAB motifs.

The FT-IR spectral studies support the information about the creation of new crystalline phases in such forms, as those revealed by the SC X-ray diffraction. The TG/DSC thermal curves indicate that complex **5** exhibits the highest stability and melting point. The thermal stability order of the obtained supramolecular adducts is as follows: L-PRO^±^·HBTA < D-PRO^±^·HBTA < DL-PRO^±^·HBTA < INA·HBTA < TPA^+^·BTA^−^. The highest stability of compound **5** is related to the deprotonated form of the HBTA component containing a carboxylate group, which is a far more powerful and demanding hydrogen bond acceptor than the original carboxylic functional. The COO^−^ group is not typically content with accepting single D−H··· COO**^−^** hydrogen bonds because it prefers to act as a multiple acceptor, especially in strong hydrogen bonds, which is reflected by the revealed network of the hydrogen bonds (Table 2) that exists in the crystal structure of TPA^+^·BTA^−^ salt (5). In this case, each of the two carboxylate oxygens act as a double acceptor, ultimately giving as many as four strong N_aminium/indole_−H··· COO**^−^** hydrogen bonds with a similar strength sustained the TPA^+^ and BTA^−^ residues. The carboxylic oxygen atoms of neutral HBTA in co-crystals 1–4 also take part in hydrogen bonding but their involvement in the creation of strong H-bonds is weaker than that in the salt, due to the fact that they act only as a single donor (zwitterionic co-crystals 1–3) or a single donor and a single acceptor (true co-crystal 4) (Table 2).

The results show that (benzylthio)acetic acid is a good building unit to be embodied into co-crystals or salts to make diversely formed, hydrogen-bonded structures with different stabilities.

## Figures and Tables

**Table 1:** A summary of crystal data and refinement details of the two-component crystals **1**–**5**.

Compound	1	2	3	4	5
Empirical formula	C_14_H_19_NO_4_S	C_14_H_19_NO_4_S	C_14_H_19_NO_4_S	C_15_H_16_N_2_O_3_S	C_19_H_22_N_2_O_2_S
Formula weight	297.36	297.36	297.36	304.36	342.44
T [K]	293 (2)	293 (2)	100 (2)	293 (2)	120 (2)
Crystal system	monoclinic	monoclinic	orthorhombic	triclinic	monoclinic
Space group	*P2_1_*	*P2_1_*	*Pna2_1_*	*P-1*	*P2_1_*
*a* [Å]	5.8620 (10)	5.8945 (15)	30.900 (2)	5.6240 (6)	9.8316 (7)
*b* [Å]	5.4076 (6)	5.4388 (10)	8.8317 (6)	8.4144 (11)	5.9747 (5)
*c* [Å]	23.570 (3)	23.773 (4)	5.2049 (4)	16.150 (2)	15.0339 (11)
*α* [°]	90.00	90.00	90.00	79.244 (11)	90.00
*β* [°]	93.052 (14)	93.06 (2)	90.00	86.751 (10)	92.051 (7)
*γ* [°]	90.00	90.00	90.00	78.586 (10)	90.00
*V* [Å^3^]	746.11 (18)	761.1 (3)	1420.40 (17)	735.85 (16)	882.54 (12)
*Z*	2	2	4	2	2
D_calc._ [g cm^−3^]	1.324	1.298	1.391	1.374	1.289
*µ* [mm^−1^]	0.229	0.225	0.241	0.231	0.197
Crystal size [mm]	0.3 × 0.3 × 0.05	0.2 × 0.2 × 0.2	0.3 × 0.2 × 0.05	0.3 × 0.2 × 0.03	0.3 × 0.05 × 0.05
*θ* range [°]	3.462–27.473	3.461–27.485	2.637–27.469	2.568–27.482	2.711–27.481
*F (000)*	316	316	632	320	364
Refl. collected/unique	5450/3199	5684/3307	10,126/3172	5756/3370	6796/3886
*R*_int_	0.0332	0.0283	0.0348	0.0199	0.0282
Observed refl. [*I* > 2*σ* (*I*)]	2375	2880	3006	2539	3605
Completeness to *θ*_max_	0.998	0.998	0.998	0.999	0.998
Goodness-of-fit on *F*^2^	1.078	1.079	1.066	1.019	0.992
*R*_1_, *wR*_2_ [*I* > 2*σ* (*I*)]	0.0583, 0.1137	0.0452, 0.1028	0.0331, 0.0752	0.0452, 0.1109	0.0375, 0.0854
*R*_1_, *wR*_2_ (all data)	0.0833, 0.1294	0.0532, 0.1107	0.0355, 0.0765	0.0639, 0.1250	0.0423, 0.0897
Largest diff. peak/hole [e Å^−3^]	0.248/−0.171	0.324/−0.171	0.288/−0.188	0.275/−0.182	0.263/−0.183
CCDC no	2212737	2212738	2212739	2212740	2212741

**Table 2:** Hydrogen bonding parameters (Å, °) in two-component crystals **1**–**5**.

D–H···A	D–H (Å)	H···A (Å)	D···A (Å)	D–H···A (°)	Symmetry Code for A
**L-PRO^±^·HBTA (1)**					
O1–H1O1···O4	1.01 (6)	1.67 (6)	2.634 (4)	159 (5)	
N1–H1N1···O3	0.88 (5)	2.38 (4)	2.938 (4)	122 (4)	−*x* + 2, *y* + ½, −*z* + 1
N1–H2N1···O3	0.96 (5)	1.92 (5)	2.848 (4)	163 (4)	*x*, *y* + 1, *z*
N1–H2N1···O4	0.96 (5)	2.55 (5)	3.265 (5)	131 (3)	*x*, *y* + 1, *z*
C10–H10A···O1	0.97	2.59	3.431 (5)	144.6	*x* + 1, *y*, *z*
C11–H11A···S1	0.97	2.94	3.885(4)	165.3	*x* + 1, *y*, *z*
**D-PRO^±^·HBTA (2)**					
O1–H1O1···O4	0.84 (4)	1.82 (4)	2.656 (3)	170 (4)	*x* + 1, *y*, *z*
N1–H1N1···O3	0.85 (4)	2.37 (3)	2.955 (3)	127 (3)	−*x* + 1, *y* − ½, −*z* + 2
N1–H2N1···O3	0.91 (3)	1.99 (3)	2.865 (3)	160 (2)	*x*, *y* − 1, *z*
N1–H2N1···O4	0.91 (3)	2.61 (3)	3.286 (3)	132 (2)	*x*, *y* − 1, *z*
C10–H10B···O1	0.97	2.63	3.459 (4)	143.9	
C11–H11B···S1	0.97	2.96	3.908 (3)	165.9	
**DL-PRO^±^·HBTA (3)**					
O1–H1O1···O4	0.90 (3)	1.71 (3)	2.606 (2)	169 (3)	
N1–H1N1···O3	0.84 (3)	2.18 (3)	2.632 (3)	113 (2)	
N1–H1N1···O4	0.84 (3)	2.20 (3)	2.892 (3)	140 (3)	−*x* + ½, *y* − ½, *z* + ½
N1–H2N1···O3	0.91 (3)	1.84 (3)	2.748 (3)	172 (3)	−*x* + ½, *y* − ½, *z* − ½
C10–H10B···O1	0.97	2.65	3.271 (3)	122.2	*x*, *y*−1, *z*
C11–H11B···O2	0.97	2.44	3.289 (3)	146.4	
**INA·HBTA (4)**					
O1–H1O1···N1	0.91(2)	1.70 (2)	2.611 (2)	175 (2)	
N2–H1N2···O3	0.92(3)	2.01 (3)	2.929 (2)	176 (2)	−*x* + 3, −*y* + 4, −*z*
N2–H2N2···O2	0.86(2)	2.12 (2)	2.965 (2)	167.6 (18)	*x*, *y* + 1, *z*
**TPA^+^·BTA^−^ (5)**					
N1–H1N1···O2	0.82(4)	2.08 (3)	2.800 (3)	146 (3)	*x*, *y*−1, *z*
N2–H1N2···O1	0.91(3)	2.00 (3)	2.869 (3)	159 (2)	
N2–H2N2···O1	0.96(3)	1.83 (4)	2.793 (3)	175 (3)	−*x* + 1, *y* + ½, −*z* + 2
N2–H3N2···O2	0.85(4)	1.93 (4)	2.776 (3)	174 (4)	−*x* + 1, *y* − ½, −*z* + 2
C2–H2B···N1	0.97	2.61	3.537 (4)	161.1	
C10–H10···O1	0.93	2.53	3.458 (3)	173.5	−*x* + 1, *y* − ½, −*z* + 2
C19–H19A···S1	0.97	3.02	3.709 (3)	129.0	−*x* + 1, *y* + ½, −*z* + 2

## Data Availability

The data presented in this article are available on reasonable request from the corresponding author. Crystallographic data have been registered at the Cambridge Crystallographic Data Centre (CCDC). The deposited CIF files were allocated the numbers 2212737 for **1**, 2212738 for **2**, 2212739 for **3**, 2212740 for **4** and 2212741 for **5**. Copies of this information may be accessed freely from the Cambridge Crystallographic Data Centre via www.ccdc.cam.ac.uk/structures (or from the CCDC, 12 Union Road, Cambridge CB2 1EZ, UK; Fax: +44-1223-336033; E-mail: deposit@ccdc.cam.ac.uk).

## Supplementary Materials

The following supporting information can be downloaded at: https://www.mdpi.com/article/10.3390/molecules27238203/s1, Table S1: Non-hydrogen bond lengths (Å) and valence angles (°) for structures **1**–**5**, Figure S1: FT-IR spectra (4000–400 cm^−1^) of L-PRO^±^·HBTA (**A**), D-PRO^±^·HBTA (**B**), DL-PRO^±^·HBTA (**C**), INA·HBTA (**D**) and TPA^+^·BTA^−^ (**E**) complexes along with their comparison with the spectral patterns registered for HBTA and respective N-containing co-former, Figure S2: TG and DSC thermograms (30–800 °C) for L-PRO^±^·HBTA (**A**), D-PRO^±^·HBTA (**B**), DL-PRO^±^·HBTA (**C**), INA·HBTA (**D**) and TPA^+^·BTA^−^ (**E**) against the TG and DSC curves registered for individual building bricks.

## Sample Availability

Samples of the compounds are not available from the corresponding author.
