# Supplementary material for: The First Noncovalent-Bonded Supramolecular Frameworks of (Benzylthio)Acetic Acid with Proline Compounds, Isonicotinamide and Tryptamine
Source: Molecules. 2022 Nov 24;27(23):8203. doi: 10.3390/molecules27238203 (PMC9740739; doi:10.3390/molecules27238203)
Supplement: Supplementary file 1 [file molecules-27-08203-s001.zip › molecules-2028994-supplementary.pdf]

molecules

Supplementary Material

The First Noncovalent-Bonded Supramolecular Frameworks of (Benzylthio)acetic Acid with Proline Compounds, Isonicotinamide and Tryptamine

Justyna Sienkiewicz-Gromiuk [1,*] and Aleksandra Drzewiecka-Antonik [2]

1 Department of General and Coordination Chemistry and Crystallography, Institute of Chemical Sciences, Faculty of Chemistry, Maria Curie-Sklodowska University in Lublin, M. Curie-Sklodowska Sq. 2, 20-031 Lublin, Poland; justyna.sienkiewicz-gromiuk@mail.umcs.pl
2 Institute of Physics, Polish Academy of Sciences, Aleja Lotnikow 32/46, 02-668 Warsaw, Poland; adrzew@ifpan.edu.pl
* E-mail address for correspondence: justyna.sienkiewicz-gromiuk@mail.umcs.pl (J. S.-G.)

molecules

MDPI

Table S1. Non-hydrogen bond lengths (Å) and valence angles (°) for structures **1-5**.

Bonds (Å)	1	2	3	Angles (°)	1	2	3
C1–O1	1.329(6)	1.333(4)	1.334(3)	O1–C1–O2	123.2(4)	123.9(3)	124.0(2)
C1–O2	1.205(5)	1.207(4)	1.203(3)	O1–C1–C2	112.9(4)	112.5(3)	112.66(19)
C1–C2	1.482(6)	1.509(4)	1.508(4)	O2–C1–C2	123.9(5)	123.6(3)	123.4(2)
S1–C2	1.801(5	1.810(4)	1.819(2)	C1–C2–S1	111.3(3)	110.9(2)	111.18(17)
S1–C3	1.803(6)	1.821(4)	1.825(3)	C2–S1–C3	100.8(3)	101.3(2)	99.29(11)
C3–C4	1.495(8)	1.514(5)	1.507(3)	S1–C3–C4	115.3(4)	115.0(3)	111.09(17)
C4–C5	1.377(8)	1.378(6)	1.387(4)	C3–C4–C5	121.1(6)	121.0(4)	120.1(3)
C4–C9	1.373(8)	1.389(5)	1.381(4)	C4–C5–C6	120.5(6)	120.9(4)	120.7(2)
C5–C6	1.377(9)	1.391(7)	1.390(4)	C5–C6–C7	121.3(7)	120.5(5)	119.9(2)
C6–C7	1.338(8)	1.365(6)	1.376(4)	C6–C7–C8	119.0(6)	119.3(4)	119.8(3)
C7–C8	1.351(9)	1.359(7)	1.384(4)	C7–C8–C9	121.0(7)	121.0(4)	120.1(3)
C8–C9	1.372(9)	1.381(7)	1.389(4)	C8–C9–C4	120.8(6)	120.8(4)	120.4(3)
N1–C10	1.491(5)	1.504(3)	1.514(3)	C3–C4–C9	121.6(6)	121.4(4)	120.9(3)
N1–C13	1.493(5)	1.504(3)	1.503(3)	C5–C4–C9	117.3(6)	117.6(4)	119.0(2)
C10–C11	1.494(6)	1.516(4)	1.521(3)	O3–C14–O4	124.9(4)	125.4(2)	126.0(2)
C11–C12	1.522(5)	1.532(4)	1.521(3)	O3–C14–C13	118.6(3)	118.6(2)	118.20(18)
C12–C13	1.544(5)	1.557(4)	1.532(3)	O4–C14–C13	116.5(3)	116.0(2)	115.8(2)
C13–C14	1.526(5)	1.540(3)	1.529(3)	N1–C13–C14	110.6(3)	110.40(19)	109.12(18)
C14–O3	1.249(4)	1.252(3)	1.250(3)	N1–C13–C12	104.9(3)	105.05(19)	103.75(17)
C14–O4	1.251(5)	1.260(3)	1.265(3)	N1–C10–C11	102.6(3)	102.6(2)	104.84(17)
				C10–N1–C13	106.7(3)	106.84(19)	108.54(16)
				C10–C11–C12	103.8(3)	103.9(2)	103.56(18)
				C11–C12–C13	105.1(3)	105.4(2)	102.99(18)
				C12–C13–C14	112.2(3)	112.1(2)	112.40(18)
**Bonds (Å)**	**4**	**Bonds (Å)**	**5**	**Angles (°)**	**4**	**Angles (°)**	**5**
C1–O1	1.297(2)	C1–O1	1.260(3)	O1–C1–O2	123.78(16)	O1–C1–O2	124.6(2)
C1–O2	1.208(2)	C1–O2	1.250(3)	O1–C1–C2	112.87(15)	O1–C1–C2	117.8(2)
C1–C2	1.501(2)	C1–C2	1.522(4)	O2–C1–C2	123.35(16)	O2–C1–C2	117.7(2)
S1–C2	1.7913(19)	S1–C2	1.809(3)	C1–C2–S1	111.50(13)	C1–C2–S1	107.60(17)
S1–C3	1.807(2)	S1–C3	1.814(3)	C2–S1–C3	101.34(9)	C2–S1–C3	99.99(12)
C3–C4	1.492(3)	C3–C4	1.508(4)	S1–C3–C4	115.21(15)	S1–C3–C4	107.67(17)
C4–C5	1.372(3)	C4–C5	1.389(4)	C3–C4–C5	120.11(19)	C3–C4–C5	120.6(3)
C4–C9	1.377(3)	C4–C9	1.385(4)	C4–C5–C6	120.8(2)	C4–C5–C6	120.3(3)
C5–C6	1.373(3)	C5–C6	1.385(4)	C5–C6–C7	120.8(2)	C5–C6–C7	120.4(3)
C6–C7	1.361(4)	C6–C7	1.378(5)	C6–C7–C8	119.4(2)	C6–C7–C8	119.7(3)
C7–C8	1.365(3)	C7–C8	1.377(5)	C7–C8–C9	119.8(2	C7–C8–C9	120.2(3)
C8–C9	1.375(3)	C8–C9	1.387(4)	C8–C9–C4	121.5(2)	C8–C9–C4	120.5(3)
N1–C10	1.326(2)	N1–C10	1.376(3)	C3–C4–C9	122.18(18)	C3–C4–C9	120.4(3)
N1–C14	1.325(2)	N1–C13	1.374(3)	C5–C4–C9	117.7(2)	C5–C4–C9	119.0(3)
C10–C11	1.373(2)	C10–C11	1.362(4)	N1–C10–C11	123.52(16)	N1–C10–C11	110.6(2)
C11–C12	1.380(2)	C11–C12	1.446(3)	N1–C14–C13	123.16(17)	N1–C13–C12	107.6(2)
C12–C13	1.377(2)	C12–C13	1.408(4)	C10–N1–C14	117.34(16)	N1–C13–C14	130.6(3)
C13–C14	1.372(3)	C13–C14	1.394(4)	C10–C11–C12	118.85(16)	C10–N1–C13	108.7(2)
C12–C15	1.500(2)	C14–C15	1.378(4)	C11–C12–C13	117.76(16)	C10–C11–C12	105.9(2)
C15–N2	1.322(2)	C15–C16	1.399(4)	C11–C12–C15	122.68(15)	C10–C11–C18	128.6(2)
C15–O3	1.227(2)	C16–C17	1.383(4)	C12–C13–C14	119.37(17)	C11–C12–C13	107.2(2)
		C12–C17	1.396(3)	C13–C12–C15	119.56(15)	C11–C12–C17	133.2(2)
		C11–C18	1.496(4)	C12–C15–N2	117.17(15)	C13–C12–C17	119.5(2)
		C18–C19	1.521(4)	C12–C15–O3	119.91(16)	C11–C18–C19	114.9(2)
		C19–N2	1.497(3)	N2–C15–O3	122.90(17)	C12–C13–C14	121.8(2)
						C12–C17–C16	118.6(3)
						C17–C16–C15	121.3(3)
						C16–C15–C14	121.1(2)
						C15–C14–C13	117.7(3)
						C12–C11–C18	125.3(2)
						C18–C19–N2	111.8(2)

molecules MDPI

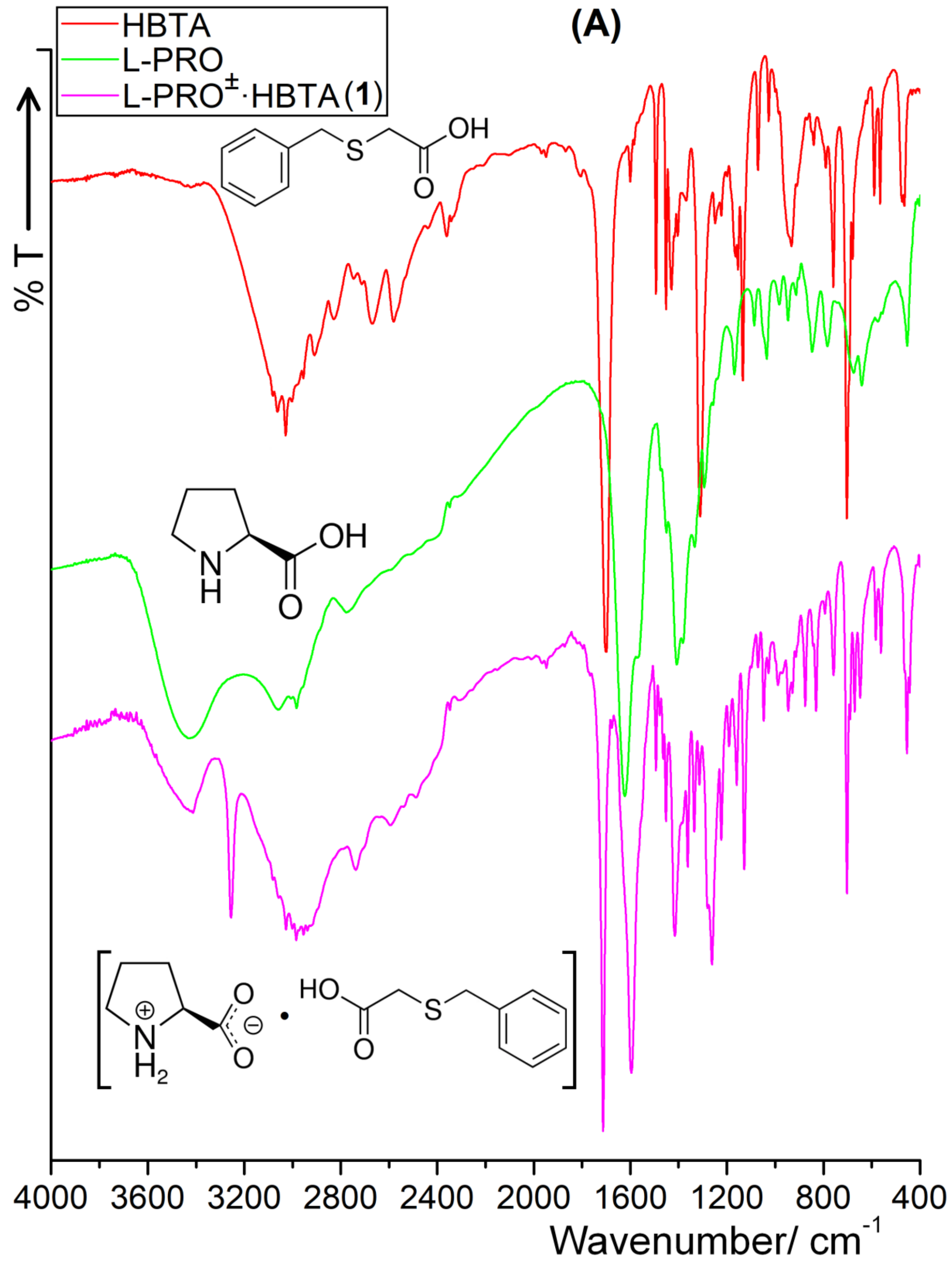

molecules

MDPI

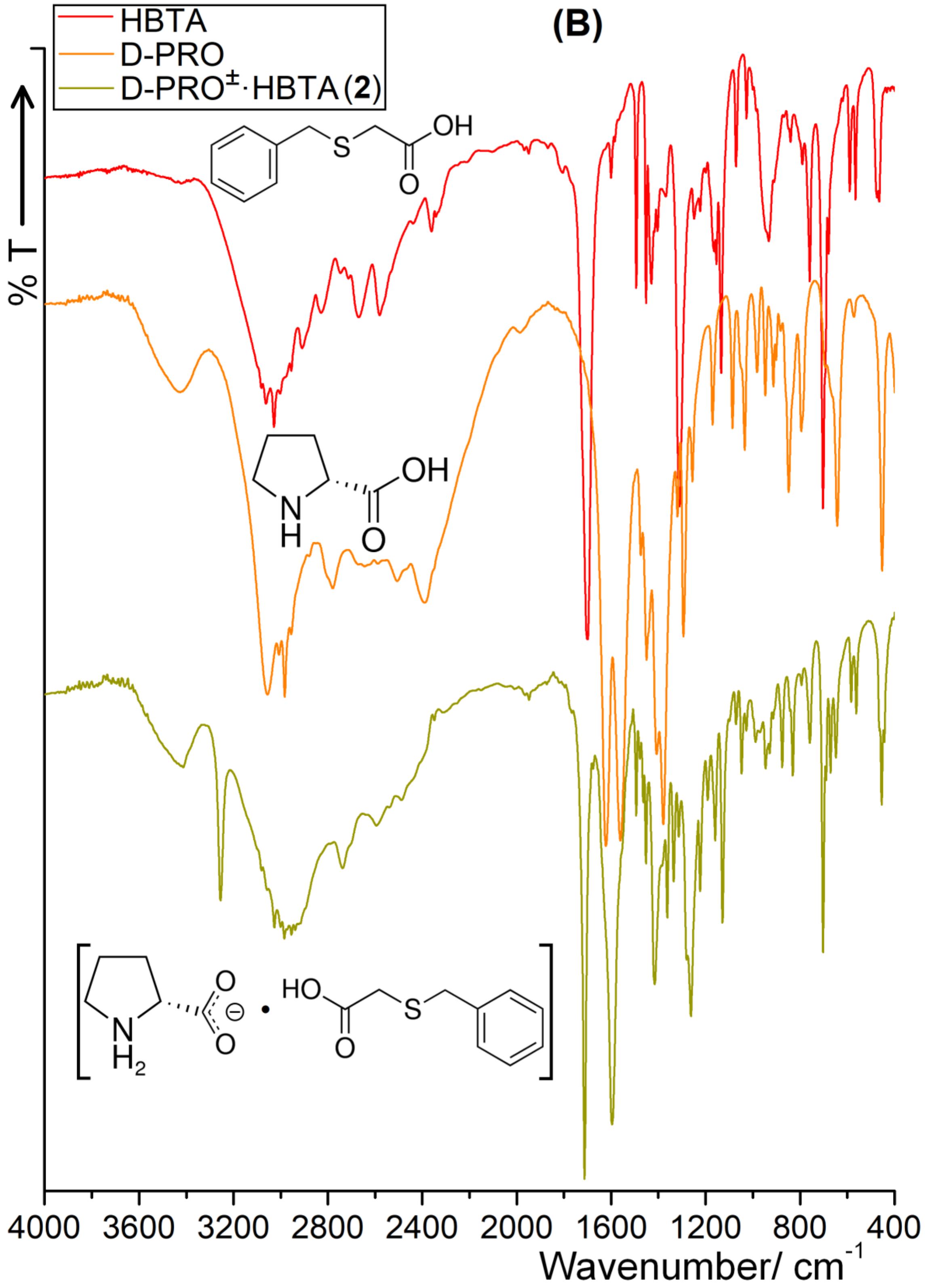

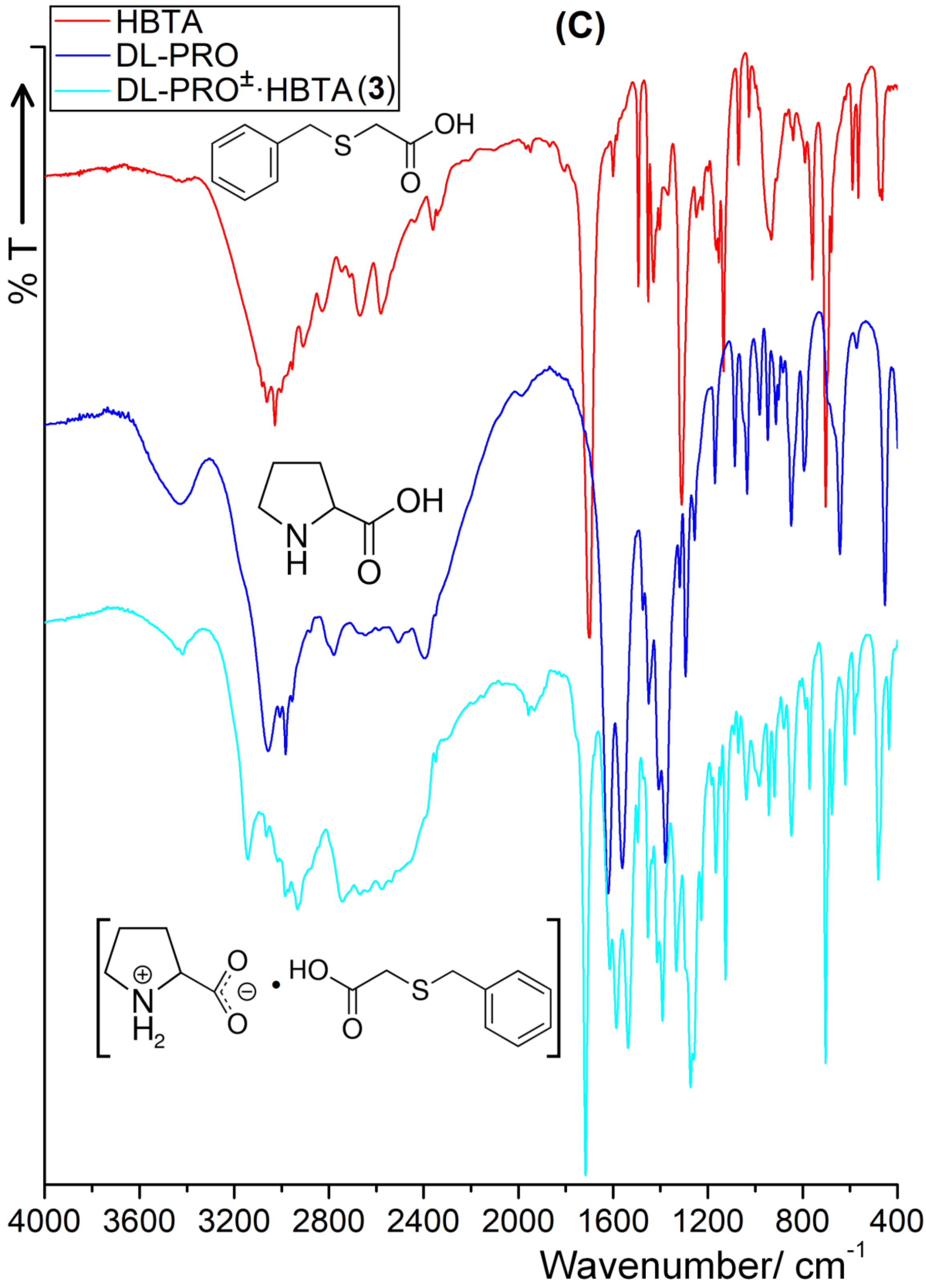
HBTA
DL-PRO
DL-PRO±·HBTA (3)
(C)
% T
OH
S
O
OH
N
H
O
O
N
H2
O
HO
O
S
4000 3600 3200 2800 2400 2000 1600 1200 800 400
Wavenumber/ cm-1

molecules MDPI

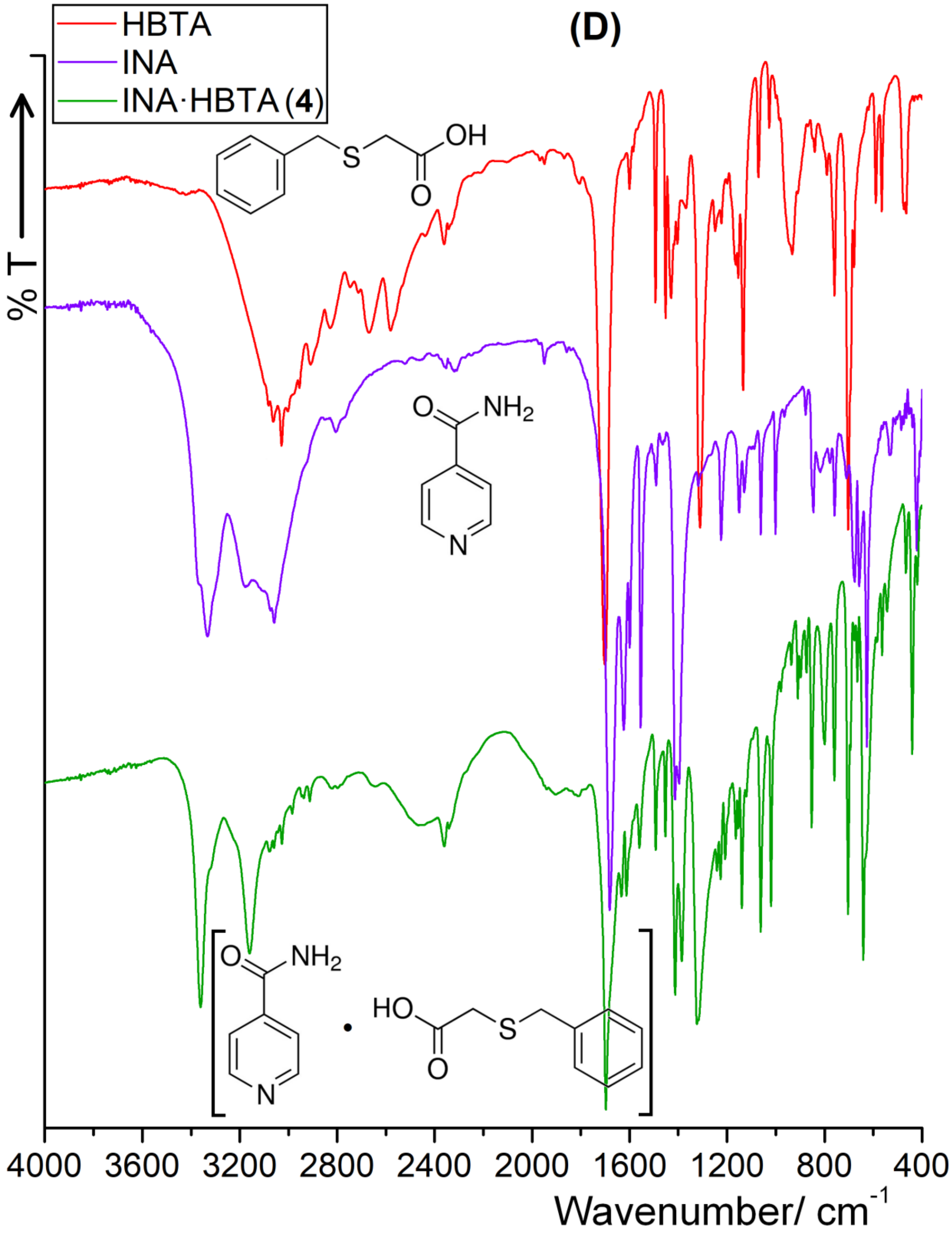

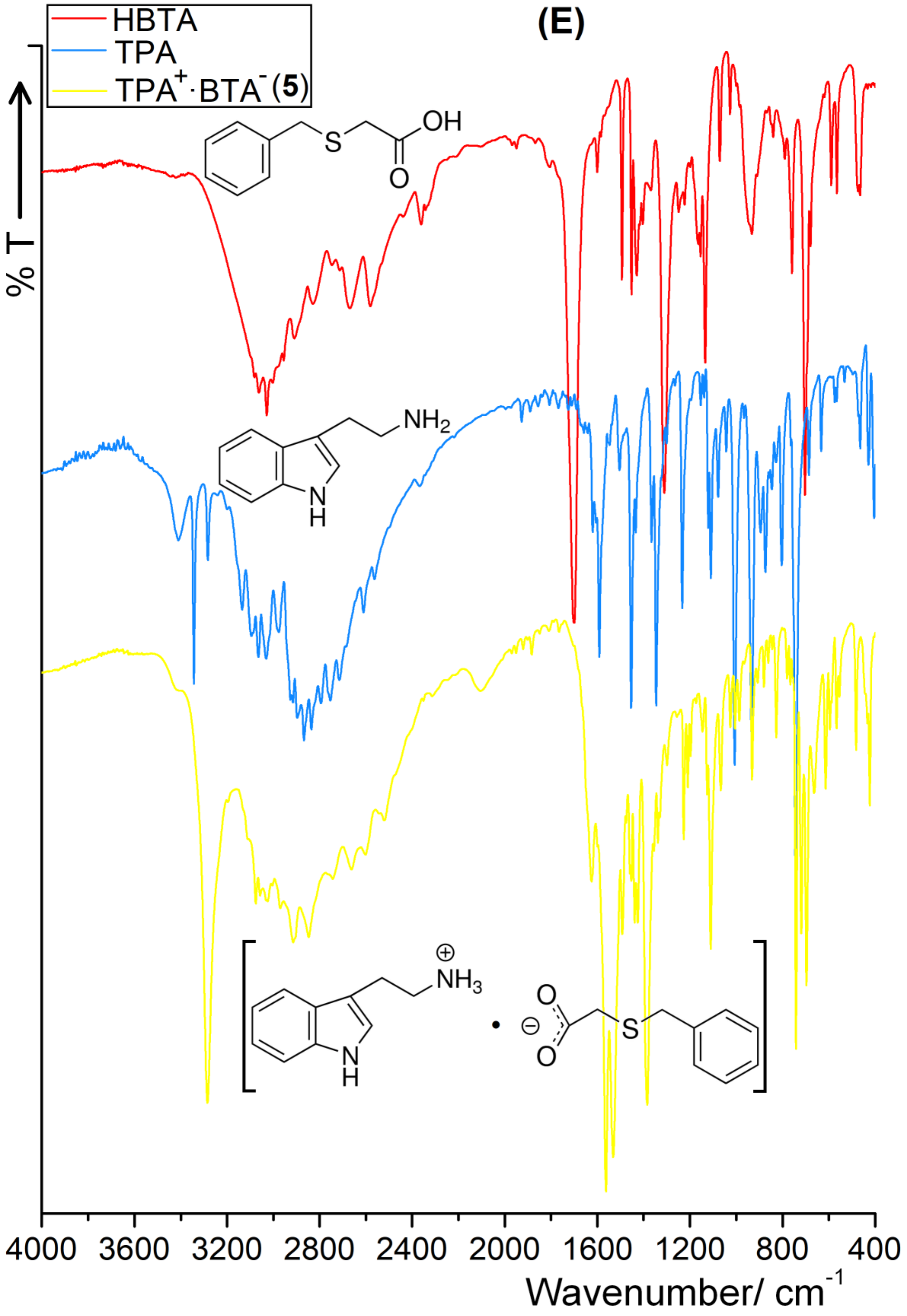

Figure S1. FT-IR spectra (4000–400 cm^{-1}) of L-PRO$^{\pm}$·HBTA (**A**), D-PRO$^{\pm}$·HBTA (**B**), DL-PRO$^{\pm}$·HBTA (**C**), INA·HBTA (**D**) and TPA^{+}·BTA^{-} (**E**) complexes along with their comparison with the spectral patterns registered for HBTA and respective N-containing co-former.

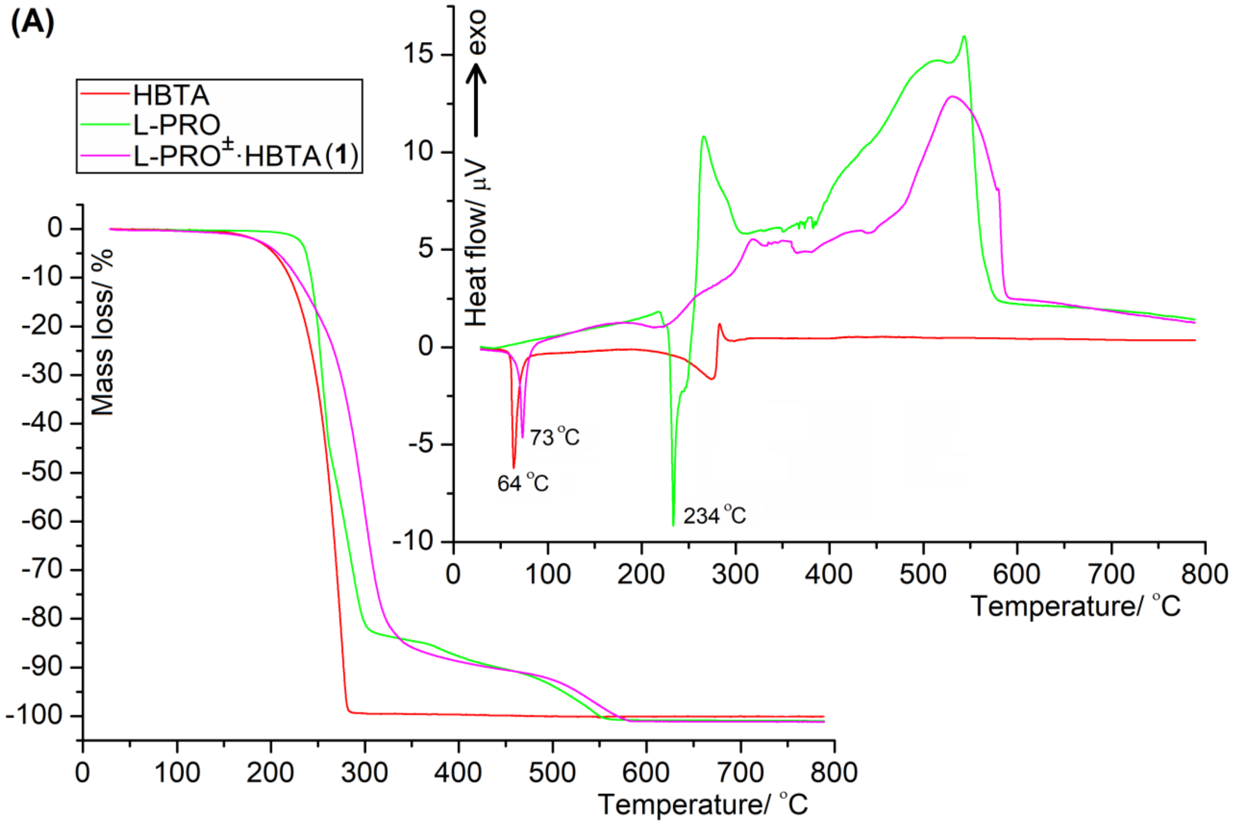
(A)
HBTA
L-PRO
L-PRO±·HBTA (1)
Mass loss/ %
Temperature/ °C
Heat flow/ µV
exo
73 °C
64 °C
234 °C

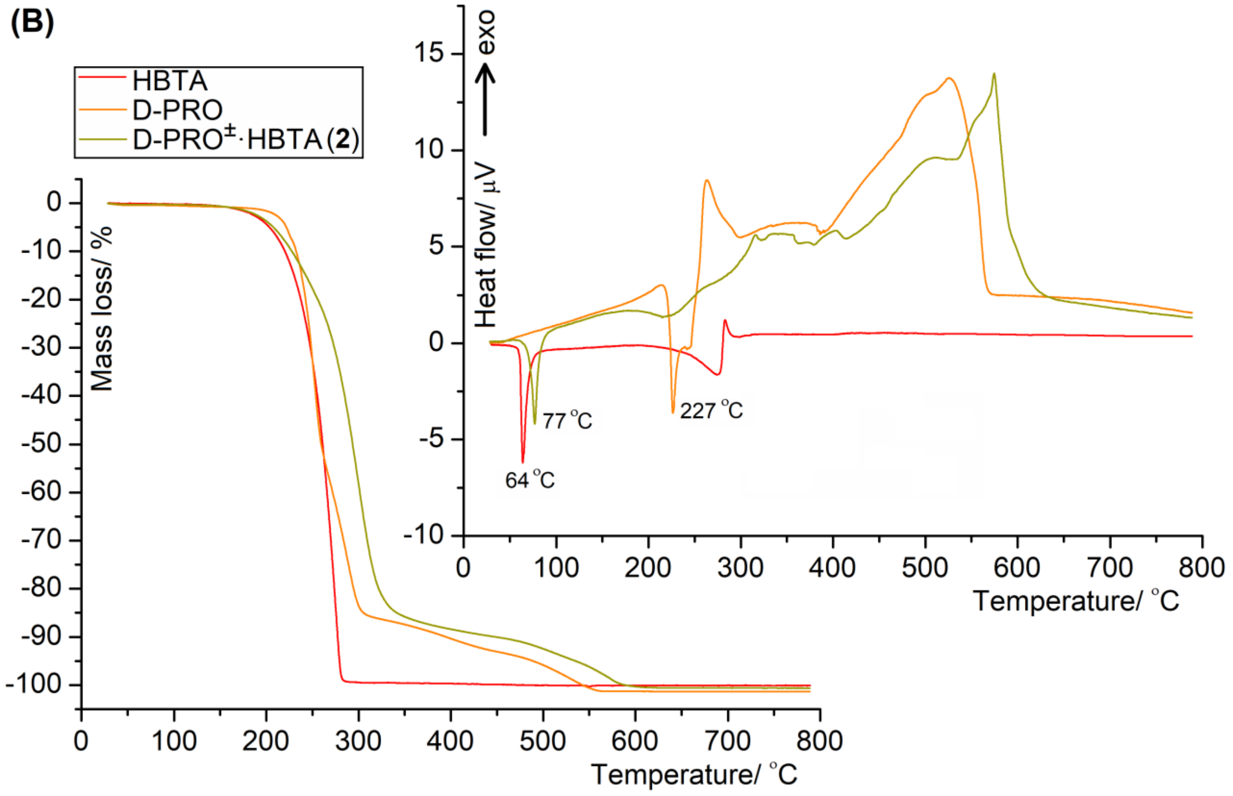
(B)
HBTA
D-PRO
D-PRO±·HBTA (2)
Mass loss/ %
Temperature/ °C
Heat flow/ µV
exo
77 °C
227 °C
64 °C

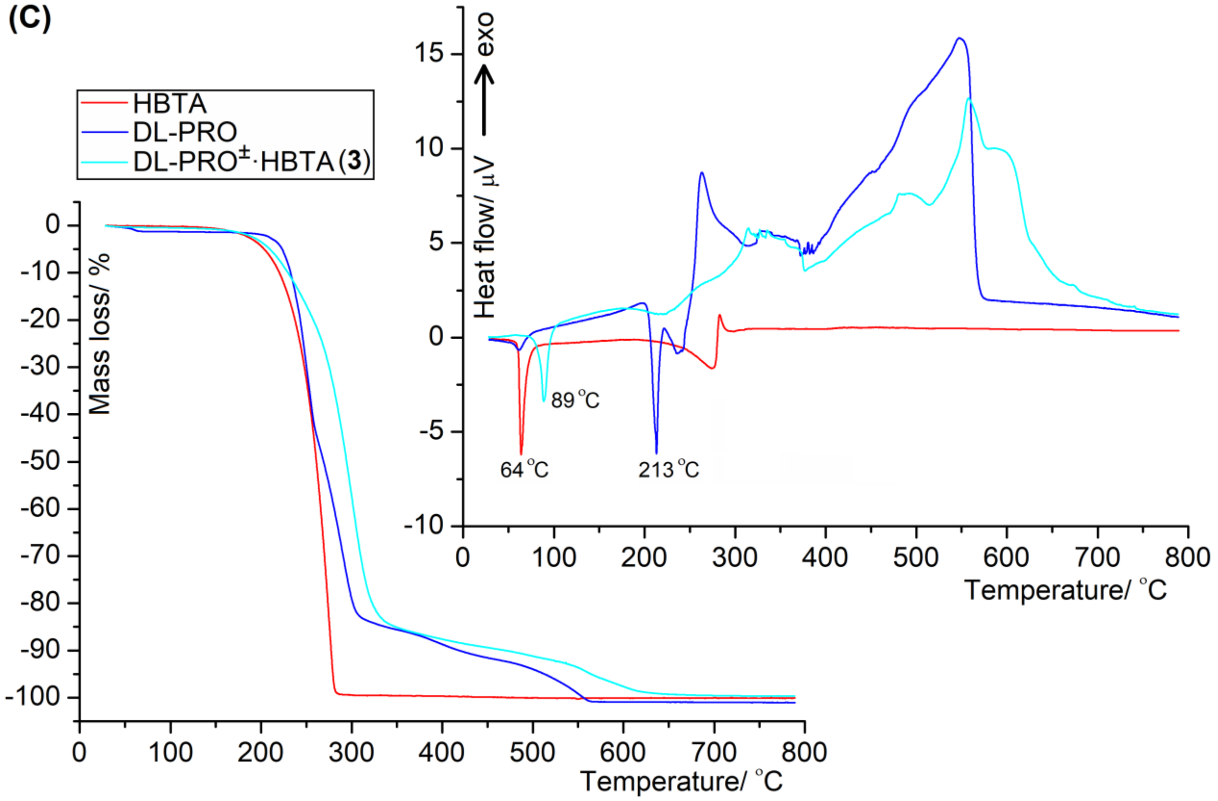
(C)
HBTA
DL-PRO
DL-PRO±·HBTA (3)
Mass loss/ %
Temperature/ °C
Heat flow/ µV
exo
64 °C
89 °C
213 °C
Temperature/ °C

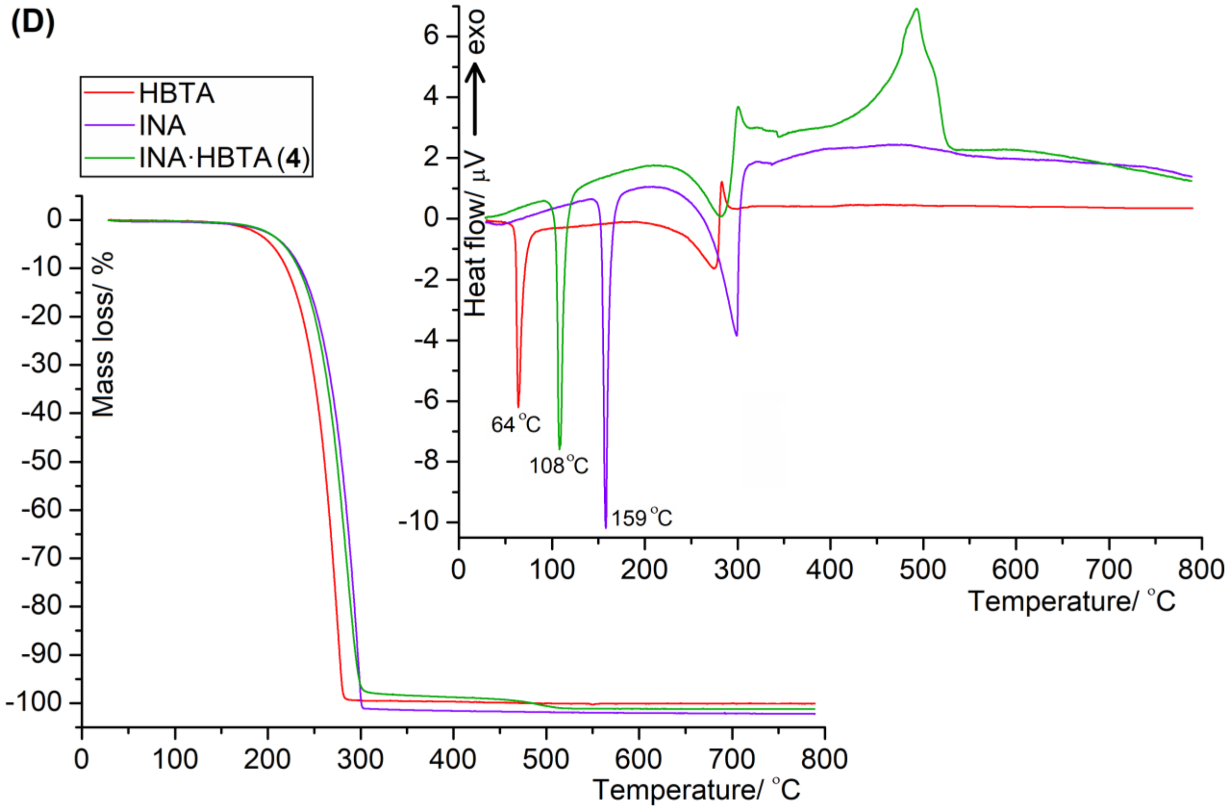
(D)
HBTA
INA
INA·HBTA (4)
Mass loss/ %
Temperature/ °C
Heat flow/ µV
exo
64 °C
108 °C
159 °C
Temperature/ °C

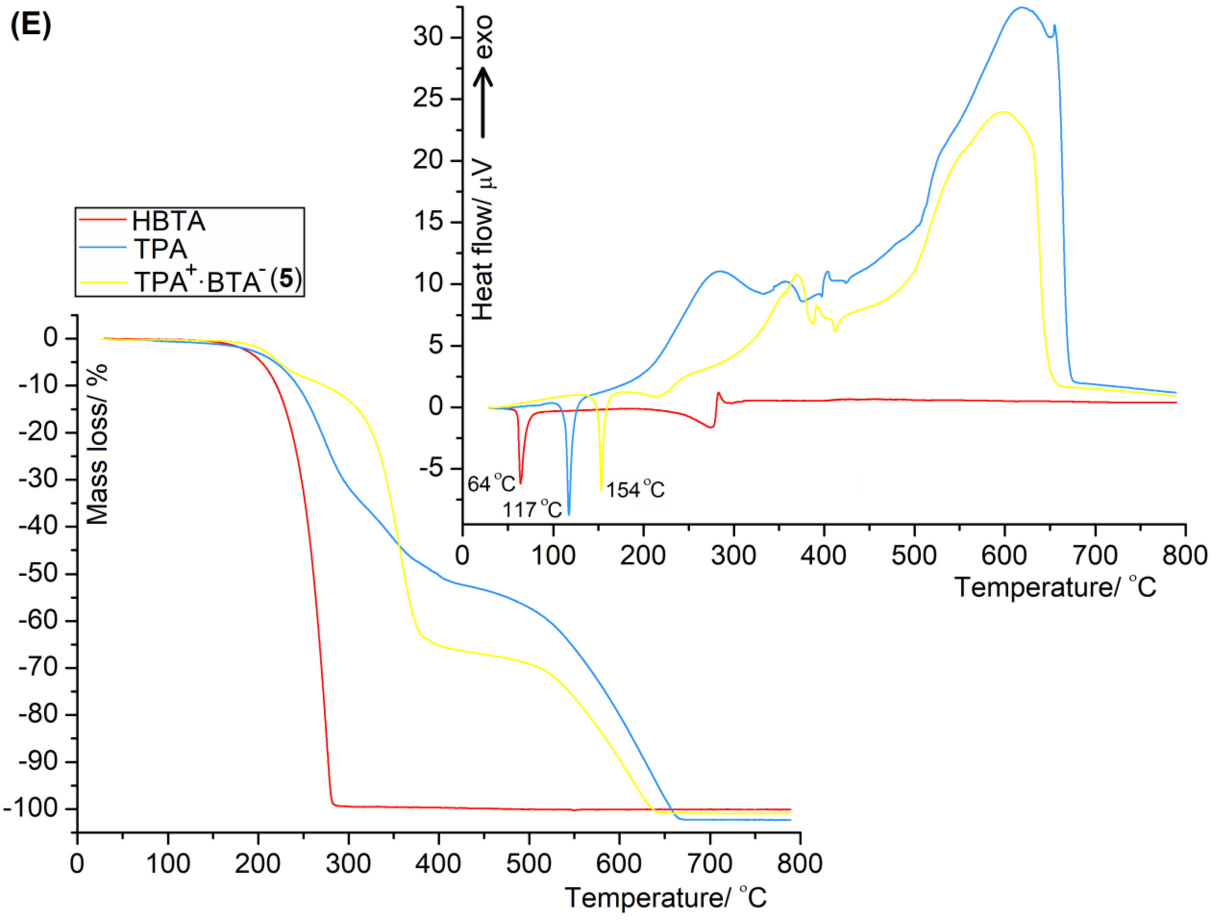

Figure S2. TG and DSC thermograms (30–800 °C) for L-PRO±·HBTA (**A**), D-PRO±·HBTA (**B**), DL-PRO±·HBTA (**C**), INA·HBTA (**D**) and TPA+·BTA- (**E**) against the TG and DSC curves registered for individual building bricks.
